# Tetrahydropyranyl Backbone Protection for Enhanced Fmoc Solid‐Phase Peptide Synthesis

**DOI:** 10.1002/chem.202501510

**Published:** 2025-07-29

**Authors:** Samuel J. Paravizzini, Craig A. Hutton, John A. Karas

**Affiliations:** ^1^ School of Chemistry The University of Melbourne Parkville VIC 3010 Australia; ^2^ The Florey 30 Royal Parade Parkville Victoria 3052 Australia

**Keywords:** amide, amino acid, protecting group, solid‐phase peptide synthesis, tetrahydropyranyl

## Abstract

Fmoc solid‐phase peptide synthesis has been indispensable for the efficient manufacture of research grade peptides and proteins, and peptide APIs. However, the solid‐phase approach is still hampered by solubility issues and aggregation of the resin‐bound peptide chain, which limits routine access to peptides > 40 amino acids in length. The use of backbone amide protecting groups, such as through the introduction of *N*‐benzyl‐based moieties and pseudoproline dipeptides, ameliorates this synthetic inefficiency somewhat. But benzyl groups can be difficult to remove postassembly, and pseudoprolines are limited to serine, threonine, and cysteine‐rich peptide segments. To enhance the utility of backbone protection, we have evaluated the tetrahydropyranyl (Thp) group as a more acid labile alternative to benzyl protection. The Thp group can be efficiently introduced to the resin‐bound peptide as a protected dipeptide and is readily cleaved and scavenged postsynthesis. A drastic improvement in the solid‐phase assembly of aggregation‐prone amyloid‐β and prion‐derived peptide fragments is observed using Thp as a backbone protecting group. We envisage that Thp‐protected dipeptides will become useful building blocks for peptide manufacturing, complementing existing backbone protecting group strategies.

## Graphical abstract

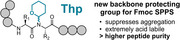

## 1 Introduction

Fluorenylmethyloxycarbonyl (Fmoc) solid‐phase peptide synthesis (SPPS) is the gold standard method for producing both research grade peptides^[^ ^1^ ^]^ and peptide APIs which are in increasing demand.^[^ ^2^ ^]^ The solid‐phase approach also enables the rapid assembly of peptide libraries, for rapid peptide‐based drug discovery and analogue development.^[^ ^3^ ^]^ Moreover, Fmoc SPPS underpins chemical protein synthesis,^[^ ^4^ ^]^ which typically involves the ligation of shorter synthetic peptides approximately 20–40 amino acids in length,^[^ ^5^ ^]^ or a single‐shot synthesis.^[^ ^6^, ^7^ ^]^ Chemical methods are important for producing homogeneous proteins bearing site‐specific post‐translational modifications,^[^ ^8^ ^]^ nonnative substitutions (e.g., d‐amino acids),^[^ ^9^ ^]^ fluorescent probes,^[^ ^10^ ^]^ and isotopic labels.^[^ ^11^ ^]^

Despite the relative success of Fmoc SPPS, it is still challenging to routinely prepare peptides > 40 amino acids in length. This is due to the iterative nature of the solid‐phase approach, which results in the accumulation of peptides that are missing one or more amino acids.^[^ ^12^ ^]^ The formation of these deletion sequences is largely caused by poor solvation and β‐sheet formation of the growing peptide chain on the solid support.^[^ ^13^ ^]^ This results in a sterically hindered N‐terminus, leading to incomplete amide couplings and Fmoc deprotections.^[^ ^14^ ^]^ As chain length increases, the synthetic failure rate increases (which is often under‐reported). Shorter peptides that are rich in aliphatic amino acids are also prone to peptide chain aggregation and poor yields.^[^ ^15^, ^16^ ^]^ This issue makes purification challenging as many deletion sequences are difficult to remove due to their physico‐chemical similarities with the target peptide.

Continuous flow^[^ ^17^ ^]^ and microwave‐assisted peptide synthesis^[^ ^7^ ^]^ have overcome this synthetic inefficiency somewhat, although a large excess of reagents is often needed, and access to specialized equipment. Alternative approaches include the use of chaotropic salts,^[^ ^18^ ^]^ highly polar solvent mixtures,^[^ ^19^ ^]^ and resins bearing solubilising tags.^[^ ^20^ ^]^ Backbone amide *N*‐protecting groups — which increase peptide chain solubility and block β‐sheet formation — have also found widespread use.^[^ ^12^, ^21^, ^22^ ^]^ Backbone protection is also effective in preventing aspartimide formation,^[^ ^23^ ^]^ suppressing aggregation in solution,^[^ ^24^ ^]^ and promoting peptide macrocyclization.^[^ ^25^ ^]^ For improving Fmoc SPPS, it is particularly effective when the protecting group is introduced at approximately every six residues.^[^ ^16^ ^]^

**Figure 1:**
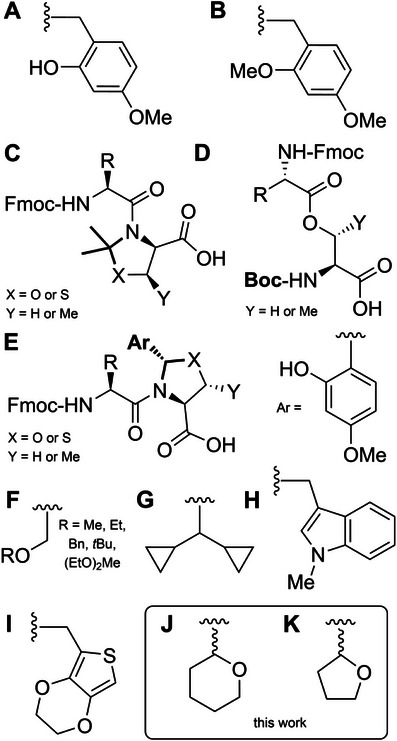
Backbone protecting groups used in Fmoc SPPS. A) 2‐Hydroxy‐4‐methoxybenzyl. B) 2,4‐Dimethoxybenzyl. C) Pseudoproline dipeptides. D) *Iso*‐acyl dipeptides. E) *N*,*O*‐Benzylidene dipeptides. F) Alkoxymethyl. G) Dicyclopropylmethyl H) 1‐Methyl‐3‐indolylmethyl. I) 3,4‐Ethylenedioxy‐2‐thienyl. J) Tetrahydropyranyl. K) Tetrahydrofuranyl.

Electron‐rich benzyl groups such as the 2,4‐dimethoxybenzyl (Dmb) group^[^ ^26^ ^]^ (Figure 1A,B) have been used widely but are sometimes difficult to cleave, and the benzylic cation that is liberated during global acid deprotection can alkylate the indole ring of tryptophan,^[^ ^27^ ^]^ and other nucleophilic amino acid side chains. Therefore, the use of multiple benzyl groups for peptides containing cysteine, methionine, and tryptophan is not recommended. The acetonide protecting group of commercially available pseudoproline dipeptides is more acid labile and less prone to alkylating amino acid side chains (Figure 1C).^[^ ^28^ ^]^ Unfortunately, the use of these building blocks is limited to serine, threonine, and cysteine‐containing peptide sequences. This limitation also applies to *iso*‐acyl,^[^ ^29^ ^]^ and *N*,*O*‐benzylidene dipeptides (Figure 1D,E).^[^ ^30^, ^31^ ^]^ Alkoxymethyl,^[^ ^32^ ^]^ dicyclopropylmethyl (Dcpm),^[^ ^33^ ^]^ indole, and thiophene‐based protecting groups^[^ ^34^ ^]^ have also been evaluated but are not widely used (Figure 1F–I).

**Scheme 1:**
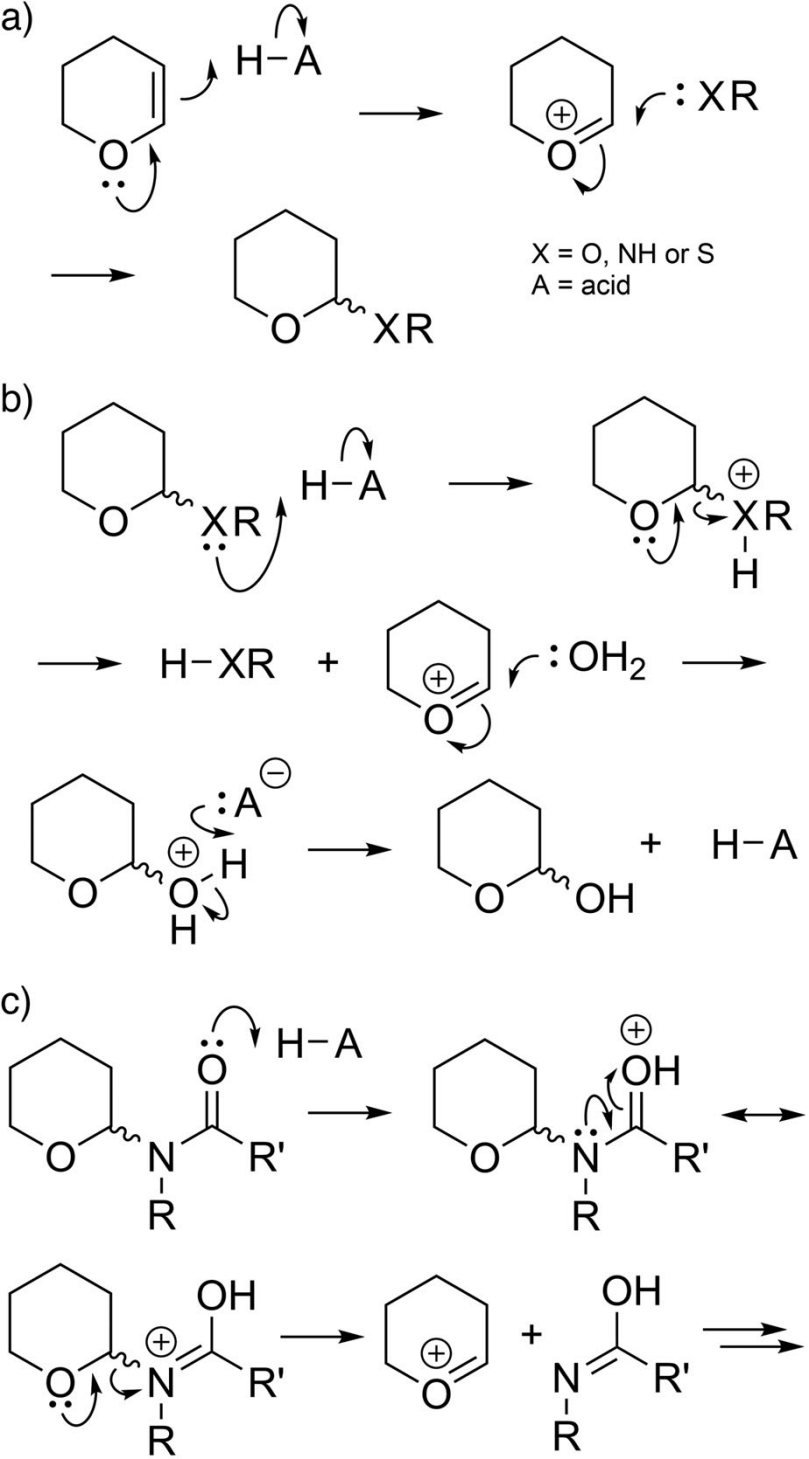
**A** Mechanism of Thp introduction. **B** Thp deprotection mechanism. **C** Plausible Thp deprotection mechanism for amides.

The ideal backbone protecting group should be: (i) synthetically accessible, (ii) highly acid labile, and (iii) universal in its application (i.e., not limited to the protection of specific amino acids). To this end, we have investigated the use of the tetrahydropyranyl group (Thp) as amide backbone protection (Figure 1J) for Fmoc SPPS. The Thp group is commonly used for hydroxyl, amine, and thiol protection;^[^ ^35^, ^36^ ^]^ it is stable under a wide range of conditions including basic conditions, and is cleaved with mild acids. Protection is achieved through the acid promoted addition of a nucleophile to the 3,4‐dihydro‐*2* *H*‐pyran double bond, forming an *X*,*O*‐acetal type linkage, where X = O, N, or S (Scheme 1A).^[^ ^37^ ^]^ This method of incorporation is milder than the introduction of linear *N*‐alkoxymethyl groups, which requires the use of corrosive reagents to prepare the required *N*‐chloromethyl intermediate.^[^ ^32^ ^]^ Acid cleavage of the Thp group is driven by protonation of the X heteroatom which allows for concomitant cleavage of the bridging acetal bond and formation of the Thp cation, which is highly electrophilic and easily scavenged by weak nucleophiles such as H_2_O (Scheme 1B). For amides, deprotection is likely to be driven by protonation of the carbonyl oxygen (Scheme 1C). The Thp group's high acid lability and stability to bases make it ideal for amide backbone protection for Fmoc SPPS. However, given its steric bulk, we chose to prepare Thp protected dipeptide precursors to enable its efficient introduction during Fmoc SPPS. We also investigated the smaller tetrahydrofuranyl (Thf) group (Figure 1K) as a less sterically hindered alternative, to potentially enhance yields in forming the tertiary amide.

## 2 Results and Discussion

### 2.1 Synthesis of Thp and Thf Protected Dipeptides

**Scheme 2:**
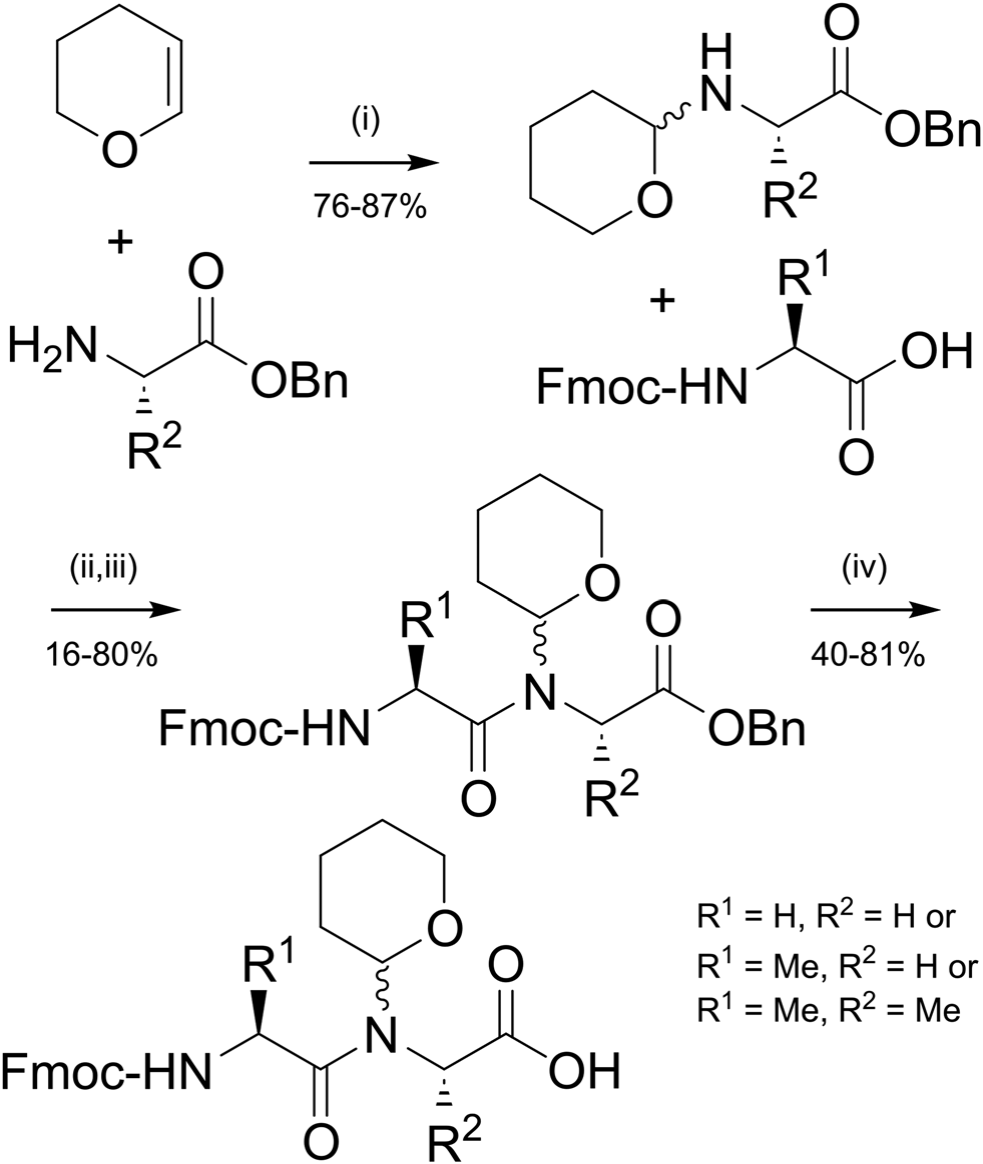
Synthesis of Thp protected Fmoc dipeptides. An analogous method is used for introducing the Thf group. **(i)** 1 N HCl_(aq)_, 1 hour. **(ii)** Fmoc amino acid activation: 2.2 eq. Fmoc‐Gly‐OH, 2.2 eq. NMM, 2.2 eq. isobutylchloroformate (IBCF) in DMF, 0 °C for 20 minutes. **(iii)** Dipeptide coupling: 1 eq. Thp‐protected amino benzyl ester, 1 eq. *N*,*O*‐bis(trimethylsilyl)acetamide in DMF (preactivated for 20 minutes), then added to the activated Fmoc amino acid. **(iv)** 10% *w/w* Pd(OH)_2_/C, H_2_ (5–15 bar), methanol, 5–24 hours.

Thp and Thf protected Fmoc dipeptides were prepared by acid‐promoted addition of 3,4‐dihydro‐*2H*‐pyran or 2,3‐dihydrofuran, respectively, to glycine or alanine benzyl esters. The *N*,*O*‐acetal intermediates are stable in 1 N HCl, and can be isolated in excellent yield (up to 87%, Scheme 2). Unfortunately (and not unexpectedly), coupling of Fmoc amino acids to the protected amino groups using uronium‐ and aminium‐based reagents^[^ ^38^ ^]^ proved unsuccessful, with only the unprotected dipeptide product isolated. This problem persisted despite modifying the reaction temperature, base additive, or solvent. Nonetheless, successful formation of the Thp and Thf protected dipeptides was achieved via the acid chloride (up to 26% yield),^[^ ^39^ ^]^ albeit with significant quantities of the unprotected dipeptide still formed. Yields could not be improved despite extensive efforts to optimise the coupling conditions (Table S1).

Therefore, we investigated other amide‐forming reaction conditions and were pleased to find that the Fmoc‐Gly‐(Thp)Gly‐OBn dipeptide could be synthesised in 61% yield using ∼2 equivalents of a mixed anhydride precursor (Table S1).^[^ ^40^ ^]^ Further improvement was achieved by utilizing *N*,*O*‐bis(trimethylsilyl)acetamide as an additive in the mixed anhydride method, which resulted in 80% isolated yield.^[^ ^41^ ^]^ Next, the benzyl esters were removed by hydrogenolysis to produce the protected dipeptide acids in 6–45% yield (over three steps). Pleasingly, significant cleavage of the Thp group was not observed using the new synthetic protocol. All glycine containing dipeptides were isolated as racemic or diastereomeric mixtures, due to the introduction of a chiral centre upon Thf/Thp introduction. Both diastereomers of Fmoc‐l‐Ala‐(Thp)l‐Ala‐OH were isolated separately, although we were unable to assign the stereochemistry of the carbon atom of the Thp group adjacent to the amide nitrogen. No epimerization of the α‐carbon was detected during the coupling reaction.

### 2.2 Deprotection Study

**Figure 2:**
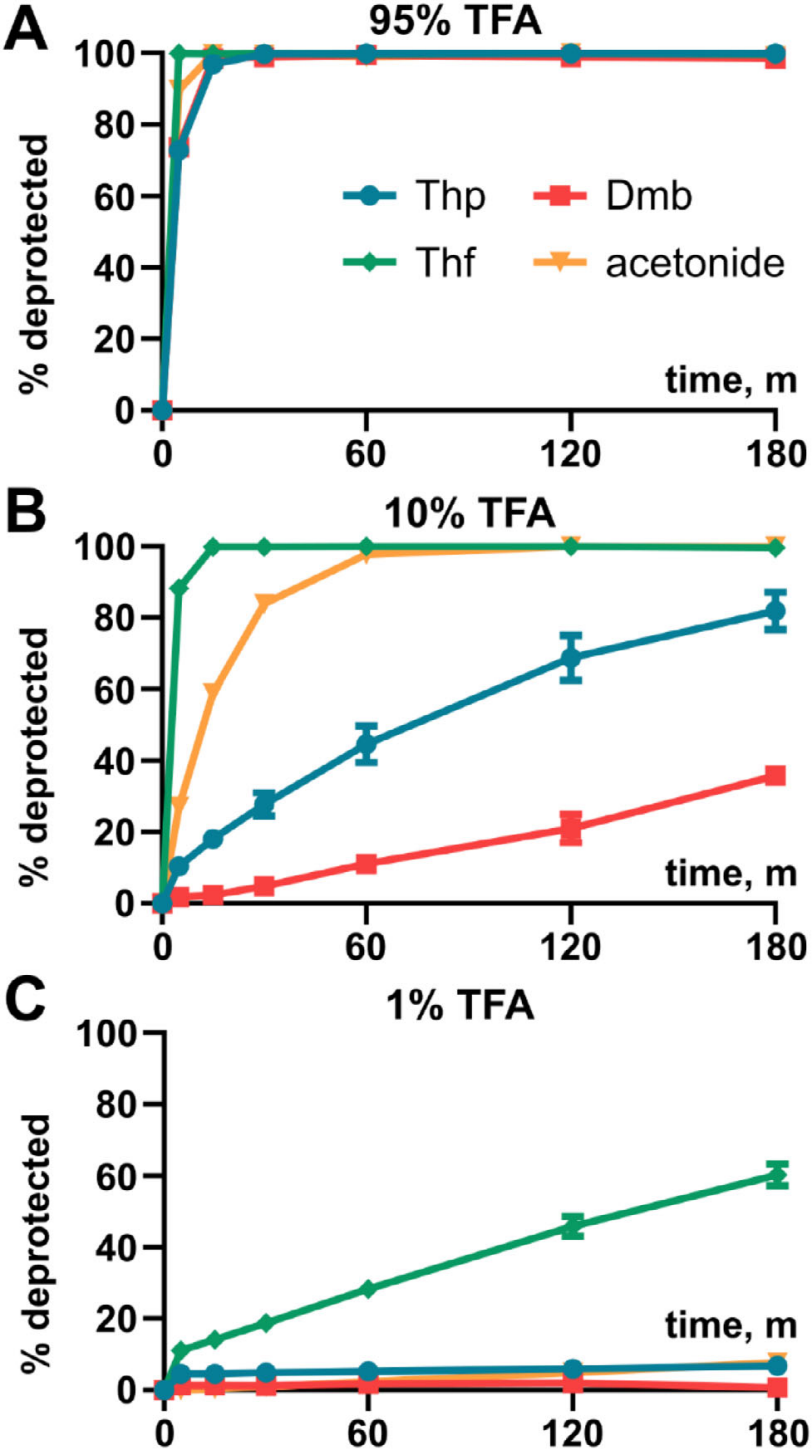
Deprotection kinetics. **A** 95% TFA, 5% H_2_O. **B** 10% TFA, 85% CH_2_Cl_2_, 5% H_2_O. **C** 1% TFA, 94% CH_2_Cl_2_, 5% H_2_O. Each time point represents three replicates. Error bars represent 1 standard deviation from the mean.

The acid lability of the Thp and Thf groups was benchmarked against the Dmb and acetonide (pseudoproline) groups, at a range of trifluoroacetic acid (TFA) concentrations. Fmoc‐Gly‐(X)Gly‐OH (where X = the protecting group) was used as a model system (except for the pseudoproline dipeptide which contains serine); water was added as the scavenger. At 95% TFA, all protecting groups were cleaved within 30 minutes, with rapid removal of the Thf and acetonide groups under these conditions (Figure 2A). To better discern the relative acid lability of each protecting group, protected dipeptides were incubated with 10% TFA. The trend in lability is: Thf > acetonide > Thp > Dmb (Figure 2B). Notably, the Thp group is significantly more acid labile than the Dmb group, which is ideal as it would reduce the likelihood of incomplete backbone deprotection during the TFA cleavage step. At 1% TFA, the Thp group is largely stable which indicates that it could be used as a solubility enhancer for protected peptide fragments (Figure 2C). In contrast, >50% of the Thf group was removed under these mild conditions. Although this high acid lability is in general desirable, the Thf protected dipeptide possesses poor bench stability, making it unsuitable as a building block for Fmoc SPPS.

In a separate study, we benchmarked the acid lability of the Thp group against the Dcpm group^[^ ^33^ ^]^ in a model system (*N*‐benzylbenzamide, Figure S1). In the ensuing kinetic studies, it was determined that the Thp group is more labile than Dcpm at both 5% and 2% TFA (Figure S2), which further indicates its suitability as a backbone protecting group. To ensure its compatibility with Fmoc SPPS, we also evaluated the Thp group's stability to 20% piperidine in *N*,*N*‐dimethylformamide (DMF), a common solution used for Fmoc deprotections. We were pleased to find that no cleavage was observed, even after 2 hours (Figure S3).

### 2.3 Epimerization Study

An additional challenge when developing new building blocks for Fmoc SPPS is to ensure that chiral integrity of the α‐carbons is maintained,^[^ ^42^ ^]^ during synthesis and solid‐phase assembly. Therefore, we coupled one of the isolated diastereomers of Fmoc‐l‐Ala‐(Thp)l‐Ala‐OH onto Rink amide resin using a standard uronium‐based coupling protocol, followed by cleavage from the solid support and Thp deprotection with TFA. The product was then analyzed via LCMS and benchmarked against the corresponding l,l, d,l, and l,d dipeptides that were prepared under standard coupling conditions. We were pleased to find that the cleavage product of the Thp protected dipeptide coeluted with the l,l standard. Moreover, no significant levels of either the d,l isomer (which would indicate epimerization during synthesis of the dipeptide), or the l,d isomer (which would indicate epimerization during activation of the dipeptide) were detected (Figure S4). This result further highlights the compatibility of Thp‐protected dipeptides with Fmoc SPPS.

### 2.4 Synthesis of Amyloid‐β(32–43)

**Figure 3:**
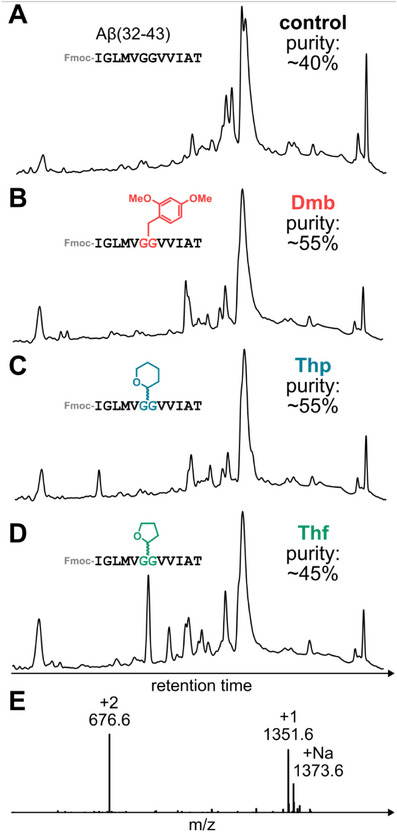
Analytical data of synthesized Fmoc‐protected Aβ(32–43). **A** Crude RP‐HPLC of the control synthesis without using backbone protection. **B** Crude RP‐HPLC of the synthesis using Dmb protection. **C** Crude RP‐HPLC of the synthesis using Thp protection. **D** Crude RP‐HPLC of the synthesis using Thf protection. **E** ESI‐MS of the major product of the Thp synthesis.

With our backbone protected dipeptides in hand, we proceeded to prepare some known challenging peptides, to determine the effectiveness of the Thp group in enhancing the efficiency of Fmoc SPPS. First, we synthesized a fragment of amyloid‐β (Aβ) peptide, which is found in Alzheimer's disease plaques in the brain.^[^ ^43^ ^]^ The C‐terminal region — Aβ(32–43) — is particularly hydrophobic, notoriously aggregation prone, and therefore challenging to assemble (Figure 3A). For comparison, we introduced Thp, Thf, and Dmb at the *N* ^α^ group of Gly^38^. We also prepared the peptide without any backbone protection as a control. Our results show that both Thp and Dmb protection significantly improved the quality of the crude peptide, compared to the control peptide (from ∼40% purity to ∼55% purity, Figure 3B–3C). Interestingly, Thf protection only provided a slight enhancement in synthetic efficiency, with several deletion sequences detected (Figure 3D). This could be due to premature cleavage of the highly labile Thf group, thereby reducing its impact on suppressing aggregation. The major product of each synthesis was confirmed by mass spectrometry (Figure 3E). These results provide strong evidence that Thp is as effective as Dmb in suppressing peptide chain aggregation.

### 2.5 Synthesis of PrP(106–126)

**Figure 4:**
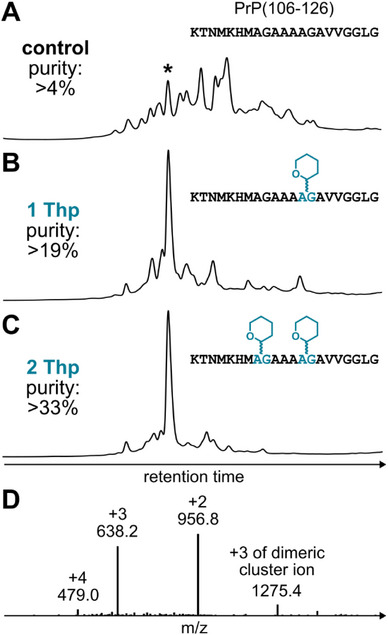
Analytical data of synthesized PrP(106–126). **A** Crude RP‐HPLC of the control synthesis without using backbone protection. **B** Crude RP‐HPLC of the synthesis using 1 Thp protecting group. **C** Crude RP‐HPLC of the synthesis using 2 Thp protecting groups. **D** ESI‐MS of the major product of the Thp synthesis.

We next applied Thp backbone protection to the synthesis of PrP(106–126) peptide, which is a fragment of the prion protein that is implicated in Creutzfeldt–Jakob disease.^[^ ^44^ ^]^ PrP(106–126) is a well‐known “difficult” sequence, and is often used as a model peptide for assessing Fmoc SPPS methodology.^[^ ^33^, ^45^ ^]^ The purity of the control synthesis was poor (∼4%, Figure 4A), which is consistent with previous studies.^[^ ^33^ ^]^ To improve assembly, we attempted to introduce the Thp group at Ala116 via Fmoc‐Ala‐(Thp)Ala‐OH, but could not isolate the resin bound precursor PrP(117–126) at sufficient purity. We next attempted an optimised assembly by protecting the *N* ^α^ group of Gly119, using Fmoc‐Ala‐(Thp)Gly‐OH. We were pleased that a significant improvement in crude purity was observed (>19%, Figure 4B), with no evidence of diketopiperazine occurring. To further enhance assembly, a second Thp group was introduced at Gly114, which resulted in > 33% crude purity (Figures 4C,D). This spectacular result highlights the value in using multiple backbone protecting groups during Fmoc SPPS, which is more feasible with highly acid labile groups such as Thp.

### 2.6 Synthesis and Isolation of Nigrocin‐HLM

Finally, Thp backbone protection was applied to the synthesis of nigrocin‐HLM, a 15‐residue α‐helical antimicrobial peptide analogue of nigrocin‐HL which derives from the skin secretion of the frog *H. latouchii*.^[^ ^46^ ^]^ We were particularly interested in determining whether Thp protection improves isolated yields. Therefore, we prepared nigrocin‐HLM — which also contains an Ala‐Gly motif — with and without Thp protection. Pleasingly, LCMS analysis indicated that crude purity increased from 32% to 54%, upon introduction of Thp during peptide assembly (Figure S5). Moreover, the isolated yield of the Thp synthesis after HPLC purification was 24% (as calculated from the synthesis scale), which compares favorably to the control synthesis (12% yield). This dramatic improvement in mass recovery further underlines the effectiveness of backbone protection in enhancing Fmoc SPPS.

### 2.7 Discussion

The solid‐phase approach^[^ ^47^ ^]^ and subsequent innovations (such as the Fmoc/*t*Bu strategy^[^ ^1^ ^]^ and microwave‐assisted SPPS^[^ ^7^, ^48^ ^]^) have revolutionised peptide synthesis. But chain assembly is still challenging, which is often due to poor solubility in DMF, and the propensity of the peptide backbone to form β‐sheets. This issue is exacerbated by the demand for longer and more complex peptides and proteins for drug development, and discovery science. Pseudoproline dipeptides have somewhat addressed the problem but cannot be used for aliphatic rich segments which are particularly prone to aggregation. Benzyl‐based groups such as Dmb can be readily used as backbone protection for glycine, and is essential for Asp‐Gly motifs to suppress aspartimide.^[^ ^45^ ^]^ But the use of multiple benzyl groups in one synthesis can result in incomplete deprotections and alkylated peptide side products, which limits its use in longer peptide segments.

The Thp group is highly compatible with Fmoc SPPS methodology, with excellent stability to piperidine. Moreover, Thp protected Fmoc dipeptides can be readily coupled to the resin bound peptide without detectable levels of epimerization. These building blocks are bench stable, and maintain high purity when stored at 4°C for > 12 months. In contrast, Thf protected dipeptides degrade significantly in < 6 months at 4°C, rendering them unviable as useful building blocks for peptide synthesis. Thp can (in theory) be used universally — much like benzyl groups — but is cleaved more efficiently due to its higher acid lability, and is easily scavenged. This is a huge advantage over Dmb as multiple sites on the peptide backbone can potentially be protected without the issue of incomplete deprotection and side reactions during the TFA cleavage step. Moreover, it is still relatively stable in 1% TFA, which suggests that it could be introduced into protected fragments to improve solubility during solution‐phase condensation reactions, or to promote peptide macrocyclization.

Synthesis of Thp protected Fmoc dipeptides can be achieved in three steps using relatively cheap and widely available starting materials. Although the final products consist of diastereomers, this has no impact on the effectiveness of Thp in Fmoc SPPS as the newly generated chiral centre is cleaved from the peptide during the TFA deprotection. However, to improve the utility of Thp‐protected dipeptides, total yields must be increased. 45% is the current benchmark (for Fmoc‐Gly‐(Thp)Gly‐OH) but there is certainly scope for further gains. The highest yield of each step (Thp protection, dipeptide formation, and hydrogenolysis) is 87%, 80%, and 81%, respectively. This suggests that optimization is feasible, particularly for the final hydrogenolysis step. Alternatively, dipeptide benzyl esters could be replaced with the corresponding allyl esters to avoid hydrogenolysis altogether, which can be conveniently deprotected and activated in one pot.^[^ ^30^ ^]^ Optimizing the coupling step of amino acids with bulkier side chains is expected to present the biggest challenge. However, the high yield obtained from using *N*,*O*‐bis(trimethylsilyl)acetamide as an additive in the coupling reaction (80%) is encouraging. A range of other racemization‐free coupling reagents, such as ynamides and allenones, could also be utilized to further improve yields.^[^ ^49^ ^]^ The preparation of Thp‐protected dipeptides containing other common amino acids such as leucine is under further investigation.

## 3 Conclusion

The Thp group has been evaluated as a backbone protecting group for peptide synthesis for the suppression of chain aggregation and consequent improved crude peptide purity. Incorporation is achieved via the preparation of bench stable, Thp‐protected Fmoc dipeptides, followed by conventional coupling methods for efficient introduction onto the solid support. Thp is substantially more acid‐labile than the Dmb group and is cleaved and scavenged efficiently. This makes it more suitable for improving the synthesis of long, “difficult” sequences that require multiple sites of backbone protection, and hydrophobic segments such as Aβ and prion fragments as we have demonstrated. The utilization of Thp also increases isolated yields, as per the synthesis and purification of nigrocin‐HLM. To improve the viability of Thp as a backbone protecting group, the introduction of other highly abundant amino acids into the dipeptide precursors will be essential. This will enable access to a wide range of challenging aliphatic‐rich peptide sequences. We anticipate that, with further synthetic optimization, Thp‐protected dipeptides will become extremely useful precursors for enhanced Fmoc SPPS, and complement existing building blocks such as pseudoproline dipeptides.

## Supporting Information

The authors have cited an additional reference within the Supporting Information.^[^ ^50^ ^]^

## Conflict of Interest

The authors declare no conflict of interest.

## Supporting information



Supporting Information

## Data Availability

The data that support the findings of this study are available in the supplementary material of this article.
