# Supplementary material for: Tetrahydropyranyl Backbone Protection for Enhanced Fmoc Solid‐Phase Peptide Synthesis
Source: Chemistry. 2025 Jul 29;31(45):e01510. doi: 10.1002/chem.202501510 (PMC12351424; doi:10.1002/chem.202501510)
Supplement: Supplementary file 1 — Supporting Information [file CHEM-31-e01510-s001.pdf]

Tetrahydropyranyl Backbone Protection for Enhanced Fmoc Solid-phase Peptide Synthesis

Samuel J. Paravizzini, Craig A. Hutton,* John A. Karas*

Supporting Information

Table of Contents

Tables, Figures and Schemes 2

Table S1 (Mixed anhydride coupling reaction optimisation) 2

Figure S1 (*N*-benzylbenzamide model systems) 3

Figure S2 (TFA kinetics of Thp dipeptides) 3

Figure S3 (Thp reactivity in 20% piperidine/DMF) 3

Figure S4 (Epimerisation study of Fmoc-Ala-(Thp)Ala-OH) 4

Figure S5 (Synthesis of nigrocin-HLM) 4

General Experimental 5

NMR Spectroscopy 7

Figure S6 (Fmoc-Ala-(Thp)Gly-OH, ^{1}H NMR) 7

Figure S7 (Fmoc-Ala-(Thp)Gly-OH, ^{13}C NMR) 8

Figure S8 (Fmoc-Gly-(Thp)Gly-OH, ^{1}H NMR) 9

Figure S9 (Fmoc-Gly-(Thp)Gly-OH, ^{13}C NMR) 10

Figure S10 (Fmoc-Ala-(Thp)Ala-OH **15A**, ^{1}H NMR) 11

Figure S11 (Fmoc-Ala-(Thp)Ala-OH **15A**, ^{13}C NMR) 12

Figure S12 (Fmoc-Ala-(Thp)Ala-OH **15B**, ^{1}H NMR) 13

Figure S13 (Fmoc-Gly-(Thf)Gly-OH, ^{1}H NMR) 14

Figure S14 (Fmoc-Gly-(Thf)Gly-OH, ^{13}C NMR) 15

Compounds 15

WILEY-VCH

Tables, Figures and Schemes

Table S1. Optimisation of the mixed anhydride conditions for the synthesis of Fmoc-Gly-(Thp)Gly-OBn. Activation of Fmoc-Gly-OH was conducted at 0°C in *N*,*N*-dimethylformamide (DMF) using isobutyl chloroformate (IBCF) and *N*-methylmorpholine (NMM) for 15 m. Then the activated ester was added dropwise into a solution of Thp-Gly-OBn (end concentration of amine is 0.05 M).

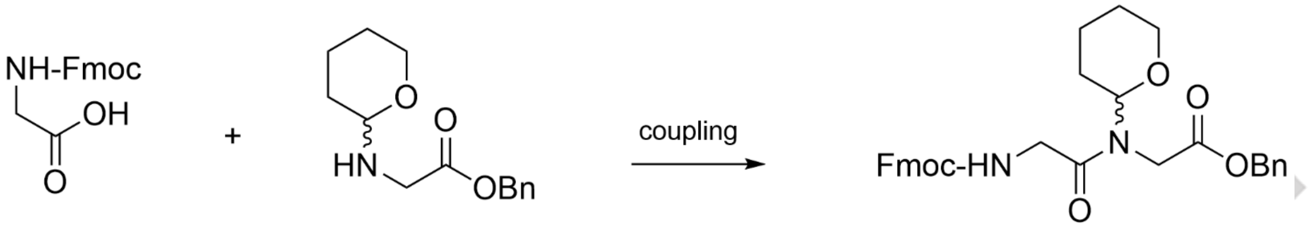

Entry	Activation	Solvent	Conditions	Yield (%)
1	Fmoc-Gly-OH (1.1 eq.), $SOCl_2$ (2 eq.), DMF (0.2 eq.)	CH_2Cl_2	0°C addition under N_2, warmed to r.t. overnight	16
2	Fmoc-Gly-OH (1.1 eq.), oxalyl chloride (1.5 eq.), DMF (0.2 eq.)	CH_2Cl_2	0°C addition under N_2, warmed to r.t. overnight	11
3	Fmoc-Gly-OH (1.05 eq.), IBCF (1.1 eq.), NMM (1.1 eq.)	DMF	0°C addition, warmed to r.t. overnight	22
4	Fmoc-Gly-OH (1.05 eq.), IBCF (1.1 eq.), NMM (1.1 eq.)	DMF	0°C addition, warmed to r.t. overnight, + NMM (2 eq.)	29
5	Fmoc-Gly-OH (1.05 eq.), IBCF (1.1 eq.), NMM (1.1 eq.)	DMF	0°C addition, warmed to 40°C overnight	25
6	Fmoc-Gly-OH (1.05 eq.), IBCF (1.1 eq.), NMM (1.1 eq.)	DMF	0°C for 6 h, r.t. overnight	45
7	Fmoc-Gly-OH (2.1 eq.), IBCF (2.2 eq.), NMM (2.2 eq.)	DMF	0°C addition, warmed to r.t. overnight	51
8	Fmoc-Gly-OH (2.1 eq.), IBCF (2.2 eq.), NMM (2.2 eq.)	DMF	0°C for 6 h, r.t. overnight	58
9	Fmoc-Gly-OH (1.4 eq.), IBCF (1.5 eq.), NMM (1.5 eq.)	DMF	0°C for 6 h, r.t. overnight	27
10	Fmoc-Gly-OH (3 eq.), IBCF (3.3 eq.), NMM (3.3 eq.)	DMF	0°C for 6 h, r.t. overnight	48
11	Fmoc-Gly-OH (2.1 eq.), IBCF (2.2 eq.), NMM (2.2 eq.)	DMF	3°C overnight	61
12	Fmoc-Gly-OH (2.1 eq.), IBCF (2.2 eq.), NMM (2.2 eq.)	DMF	-22°C overnight	33
13	Fmoc-Gly-OH (2.1 eq.), IBCF (2.2 eq.), NMM (2.2 eq.)	DMF	3°C overnight, + *N*,*O*-*bis*(trimethylsilyl)acetamide (1 eq.)	80

[a] Yields are reported as isolated yields, [b] are reported as LCMS yields [c] r.t. = room temperature.

WILEY-VCH

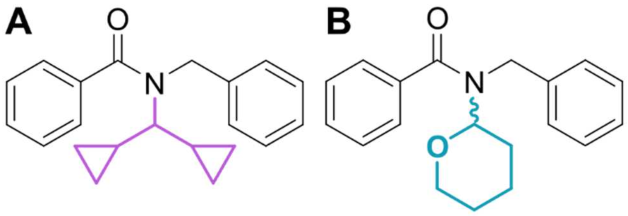

Figure S1. *N*-benzylbenzamide model system. **A** Dcpm protected. **B** Thp protected.

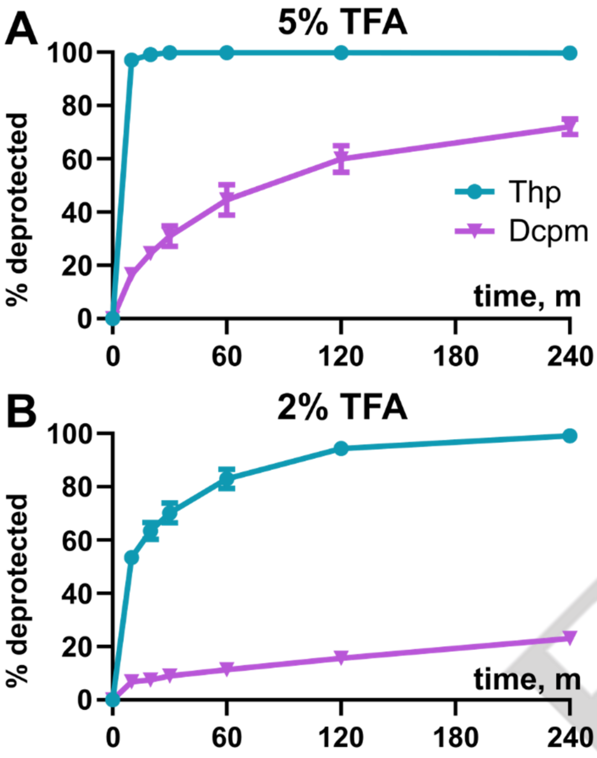

Figure S2. Cleavage kinetics of Dcpm and Thp. **A** 5% TFA, 90% CH_2Cl_2, 5% H_2O. **B** 2% TFA, 93% CH_2Cl_2, 5% H_2O. Each time point represents 3 replicates. Error bars represent 1 standard deviation from the mean.

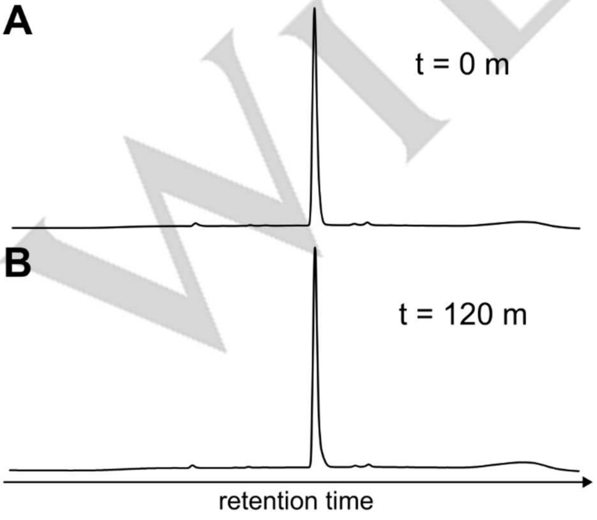

Figure S3. Incubation of Thp protected *N*-benzylbenzamide (**Figure S1B**) in 20% piperidine/DMF. **A** Time = 0 m. **B** Time = 120 m.

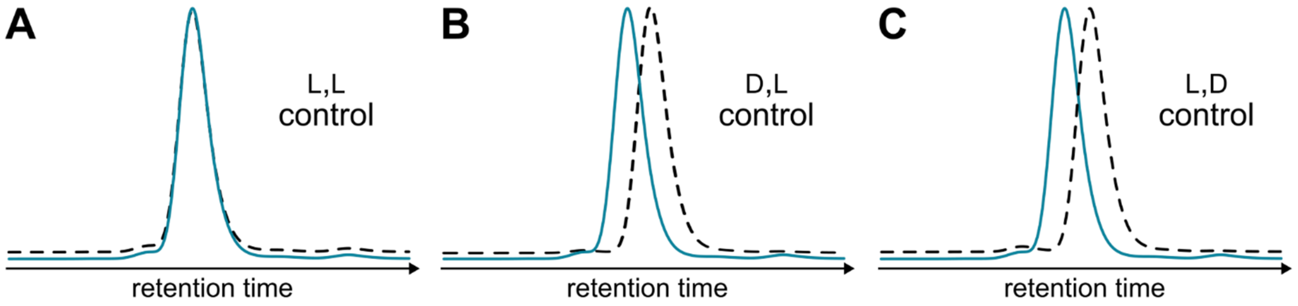

Figure S4. Epimerisation study of Fmoc-Ala-(Thp)Ala-OH dipeptides via LCMS analysis with control dipeptides (dotted lines). **A** L,L control. **B** D,L control. **C** L,D control.

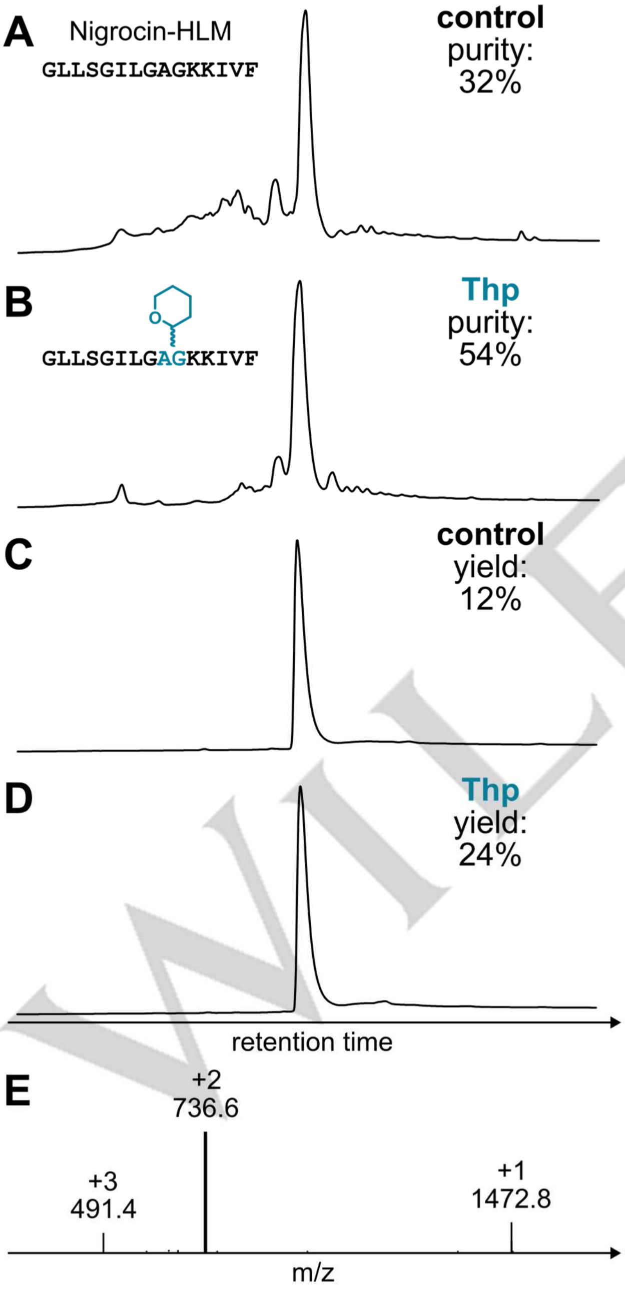

Figure S5. Synthesis of nigrocin-HLM. **A** Crude RP-HPLC of the control synthesis without using backbone protection. **B** Crude RP-HPLC of the synthesis using Thp backbone protection. **C** Pure RP-HPLC of the control synthesis without using backbone protection. **D** Pure RP-HPLC of the synthesis using Thp backbone protection. **E** ESI-MS of purified nigrocin-HLM from the Thp synthesis. The ESI-MS of the control synthesis was comparable. Both syntheses were prepared on 25 µmol scales, with 10.7 mg of purified peptide obtained from the Thp synthesis (24% isolated yield), and 5.5 mg obtained from the control synthesis (12% isolated yield).

General Experimental

Fmoc protected amino acids were purchased from Combi-Blocks (U.S.A.) and Mimotopes Pty Ltd (Australia), 1-[Bis(dimethylamino)methylene]-1H-1,2,3-triazolo[4,5-b]pyridinium 3-oxide hexafluorophosphate (HATU) and (1-Cyano-2-ethoxy-2-oxoethylidenaminooxy)dimethylamino-morpholino-carbenium hexafluorophosphate (COMU) were purchased from Combi-Blocks (U.S.A.), dicyclopropylmethylketone, Ti(iOPr)$_4$, $NaBH_4$ and triisopropylsilane (TIS) were purchased from AK scientific (U.S.A), N,N-diisopropylcarbodiimide (DIC), diisopropylethylamine (DIEA), NMM, IBCF, benzylamine, 3,4-dihydro-2(*H*)-pyran, 2,3-dihydrofuran, trifluoroacetic acid (TFA), BF_3.Et_2O, thionyl chloride, oxalyl chloride, acetonitrile and $Pd(OH)_2$/C were purchased from Merck (Australia), Oxyma was purchased from CEM peptides (U.S.A) and DMF, ethyl acetate, hexane, CH_2Cl_2, diethyl ether, methanol, ethanol, acetone, 2,2-dimethoxypropane, isopropanol, NaOH, NaCl, $MgSO_4$, Na_2SO_4, acetic acid, $NaHCO_3$ and HCl were purchased from both Thermo Scientific (U.S.A) and ChemSupply (Australia.). Reactions were monitored using thin layer chromatography (TLC) on commercial silica gel 60 aluminium-backed plates coated with fluorescent indicator F254, purchased from Merck. Plates were visualised under UV light (254 nm) alone or in conjunction with potassium permanganate, phosphomolybdic acid or ninhydrin-based stains. Peptides were assembled manually on Wang, Rink amide or 2-chlorotrityl chloride resin purchased from ChemPep (U.S.A), all Fmoc amino acids were coupled with HATU/DIEA or DIC/Oxyma/DIEA. All compounds were synthesised from the purchased reagents, then purified and characterised through the following. Silica gel used was Davisil Chromatographic Silica Media LC60A 40-63 micron, with solvent systems as specified. Flash chromatography with solvent systems specified below was performed on the Shimadzu Biotage Isolera prime system (John Morris Group, Australia).

NMR spectra were recorded on a Bruker Avance III 600 instrument with operating frequencies of 600 MHz for ^{1}H NMR and 161 MHz for ^{13}C NMR (Bio 21 institute, University of Melbourne) and a Bruker 400 instrument with operating frequencies of 400 MHz for ^{1}H NMR and 101 MHz for ^{13}C NMR (University of Melbourne). Chemical shifts (δ) were expressed in ppm, with residual undeuterated solvent peaks used as an internal reference (DMSO-d_6 ^{1}H NMR = 2.50 ppm, ^{13}C NMR = 39.52 ppm; $CDCl_3$ ^{1}H NMR = 7.26 ppm, ^{13}C NMR = 77.16 ppm). The following letters assign NMR peak multiplicities: s = singlet, d = doublet, t = triplet, q = quartet, p = pentet, m = multiplet, br s = broad singlet, br m = broad multiplet, dd= doublet of doublets, dt = doublet of triplets, td = triplet of doublets, dtt = doublet of triplet of triplets, dq = doublet of quartets, qd = quartet of doublets, app. td = apparent triplet of doublets. Split peaks caused by diastereomers or rotamers (rotational restriction) are denoted as X/X ppm.

High resolution mass spectra (HRMS) were collected via electrospray ionisation (ESI) mass spectrometry, using the Thermo Scientific Exactive Plus Orbitrap mass spectrometer (Bio 21 institute, University of Melbourne) and liquid chromatography mass spectra (LCMS) were collected via electrospray ionisation (ESI) mass spectrometry, using the Agilent 1260 infinity II LC/MSD (University of Melbourne). Buffer A = 0.05% TFA in water; buffer B = 0.05% TFA in acetonitrile. The standard method used was either 5-95% buffer B over a 7 m gradient for small molecules or 5-65% buffer B over a 12 m gradient for larger peptides on a Dr Maisch C18 analytical column (0.4 mL/min). Purification was conducted on an Agilent 120 LC system using the same buffer system, and a C18 Phenomenex column (5 μm, 110 Å, 15 mL/min), 150 x 21.2 mm.

2.2 Synthesis of Dcpm-benzylamine

Benzylamine (1 eq., 2 mmol), trimethylsilyl-2-pyrrolidinone (2.5 eq., 5 mmol), dicyclopropylmethylketone (1 eq., 2 mmol) and Ti(iOPr)$_4$ (1 mol%) were combined neat at 70°C under N_2 atmosphere for 6 h. The mixture was cooled to room temperature, then ethanol was added, followed by $NaBH_4$ (3 eq., 6 mmol) and the mixture stirred in an open atmosphere overnight. The mixture was diluted with H_2O and the ethanol was evaporated. The product was extracted into $CHCl_3$, and the organic layer washed with H_2O, dried over $MgSO_4$, and then evaporated. The crude mixture was purified by column chromatography using CH_2Cl_2/5% methanol + TEA (0.1% v/v) as an eluent.

2.3 General Procedure for Pyran/Furan Protection

3,4-Dihydro-2(*H*)-pyran or 2,3-dihydrofuran (1 eq., 11 mmol) was stirred vigorously in 1N $HCl_{(aq)}$ (4 - 6 mL) at 0°C for 15-30 m until a homogenous solution was obtained. This was added to a 0°C solution of an amino acid ester (1 eq., 11 mmol, in 4-6 mL 1N HCl) and then stirred at room temperature for 1 hr. The solution was neutralised with $NaHCO_3$ (sat.) until pH >7, then extracted with ethyl acetate (×3). The organic layer was washed with H_2O, dried over $MgSO_4$ and then evaporated to leave the protected amino acid as a colourless oil. If purification was necessary, it is specified below.

2.4 General Method for Peptide Coupling Reactions

a. HATU or COMU Coupling

Fmoc-Xaa-OH (1.2 eq., 1 mmol) and HATU or COMU (1.2 eq., 1 mmol) were dissolved in dry DMF (5 mL) and cooled to 0°C, to which DIEA (3 eq., 3 mmol) was added. After 5 m, it was added dropwise to a solution of the amino acid (1 eq., 1 mmol) in DMF (5 mL), then warmed to room temperature. The mixture was stirred overnight,

then diluted with H_2O (75 mL) was added and then the mixture extracted into ethyl acetate (x 3). The organic layer was washed with 5% HCl (aq.), sat. $NaHCO_3$ (aq.) and then brine, dried over $MgSO_4$ and evaporated to leave the crude product. This was purified by recrystallisation or by column of flash chromatography.

b. Acid Chloride Coupling

Fmoc-Xaa-OH (1 eq., 2 mmol) was suspended in dry CH_2Cl_2 (50 mL) with DMF (0.2 eq.) at 0°C under a N_2 atmosphere. Then, $SOCl_2$ (2 eq., 4 mmol) or oxalyl chloride (1.1 eq., 2.2 mmol) was added, and the solution stirred at 0°C for 3 h until clear. The CH_2Cl_2 was then evaporated and the crude dissolved in ethyl acetate. The organic layer was washed with H_2O and brine, dried over $MgSO_4$ and then evaporated under N_2 to reveal the acid chloride as a white to yellow-green solid that was used immediately without further purification. The acid chloride (1 eq., 5 mmol) was then dissolved in dry CH_2Cl_2 (50 mL) and added dropwise to a solution of the amino acid (1 eq., 5 mmol) in dry CH_2Cl_2 (30 mL) with DIEA (1 - 5 eq., 2–10 mmol) at 0°C under N_2 atmosphere. The mixture was stirred at room temperature under N_2 for 2 days, then washed with 0.5 N HCl, H_2O (l) and sat. $NaHCO_3$ aq., dried over $MgSO_4$ and evaporated to yield the crude product mixture which was purified by flash chromatography.

c. Acid Chloride Coupling for Model System

Benzoyl chloride (1.1 eq., 0.55 mmol) was suspended in dry CH_2Cl_2 (5 mL) and added dropwise to a solution of the amine (1 eq., 0.5 mmol) in dry CH_2Cl_2 (5 mL) with DIEA (1.1 eq, 0.55 mmol). The mixture was then stirred at room temperature under N_2 for 2 days, then washed with 0.5 M HCl, H_2O (l) and HCO_3^- (5% w/v, aq.), dried over $MgSO_4$, filtered, and evaporated to produce the crude product mixture. The filtrate is then evaporated, and the crude mixture purified by flash chromatography.

d. Mixed Anhydride Coupling

Fmoc-Xaa-OH (2.1 eq.) was dissolved in DMF (5 mL) at 0°C. *N*-methylmorpholine (2.2 eq.) and isobutyl chloroformate (2.2 eq.) was then added and the mixture stirred for 20 m. Simultaneously, the protected amino acid (1 eq.) and N,O-bis(trimethylsilyl)acetamide (1 eq.) were dissolved in DMF (0°C) and stirred for 20 m. The activated amine was then added to the activated ester and the mixture was left at 3°C overnight. It was then diluted with ethyl acetate (50 mL) and washed with 0.5 N HCl, sat. $NaHCO_{3(aq.)}$ and brine. The organic layer was dried over Na_2SO_4 and evaporated, with the crude product being purified by flash chromatography.

2.5 General Procedure for Hydrogenolysis of Benzyl Ester

The benzyl ester (1 eq., 0.5 mmol) was dissolved in methanol and $Pd(OH)_2/C$ (10 - 20 mol%) was added to the solution. The reaction was stirred at room temperature under a H_2 atmosphere (5-15 bar) for 5-16 h, after which it was filtered. The methanol was evaporated, and the crude product was purified by flash chromatography and then lyophilised to form a dry foam.

2.6 General Procedure for *t*Bu Ester Cleavage

The *tert*-butyl ester (1 eq., 1.5 mmol) was dissolved in 50% TFA/ CH_2Cl_2 and stirred at room temperature until complete consumption of the starting material was observed; (9.5 : 0.5 CH_2Cl_2 : methanol + 0.1% TEA). The solvent was then evaporated, and the product was lyophilised to produce the free acid as a dry foam.

2.7 Esterification of Amino Acids

An amino acid (1 eq., 5 mmol) is dissolved in an alcohol (50 mL) and thionyl chloride (1.5 - 2 eq., 7.5 - 10 mmol) is added dropwise. The solution was stirred at room temperatures for 4-24 h until the starting material was consumed; TLC (9.5 : 0.5 CH_2Cl_2 : methanol + 0.1% TEA). The solvent was evaporated to leave the esterified amino acid as the hydrochloride salt.

2.8 Procedure for Pseudoproline Formation

Fmoc-Gly-Ser-OEt (1 eq., 0.5 mmol) was dissolved in acetone : 2,2-dimethoxypropane (1 : 1) at 0°C and then $BF_3.Et_2O$ (1.1 mol%) was added. The solution was stirred at room temperature for 6 h before the solvent was evaporated. Ethyl acetate was added and the organic layer washed with sat. $NaHCO_{3(aq.)}$, H_2O and then brine, dried over $MgSO_4$ and evaporated to reveal the crude mixture. This was then purified via flash chromatography.

2.9 General Procedure for Ester Hydrolysis in the Presence of Fmoc[50]

Fmoc-Xaa-(PG)Yaa-OR (R = Me or Et) (0.04 M, 1 eq., 0.3 mmol) was dissolved in iPrOH/H_2O (7 : 3 with 0.8 M $CaCl_2$) and 1N NaOH (1.2 – 2 eq., 0.4 - 0.6 mmol) was added. The reaction was stirred at room temperature for 1-16 h, being monitored by TLC (1 : 1 ethyl acetate : hexane + 0.1% AcOH). It was then acidified with 1N HCl (till pH <5) and the mixture extracted with ethyl acetate (×3), which was dried with $MgSO_4$ and evaporated; the crude product purified via flash chromatography.

2.10 TFA Kinetics

The amide protected compound (0.05 - 0.1 mmol) was dissolved in 2 mL of 1-95% TFA in CH_2Cl_2 and stirred at room temperature. 20 µL aliquots were taken at t = 0, 5, 15, 30, 45 and 60, 120 and 180 mins, being neutralised with $NaHCO_{3(aq)}$ buffer (380 µL), dissolved in acetonitrile (400 µL) and then analysed by LCMS.

WILEY-VCH

2.11 General SPPS Procedure

Polypeptides were synthesised on either 2-chlorotrityl chloride, Wang, or Rink amide resin by Fmoc SPPS. The resin was loaded with Fmoc-Xaa-OH using DIEA (8 eq.) in CH_2Cl_2 for the 2-chlorotrityl chloride resin, coupled with DIC (6 eq.) and dimethylaminopyridine (DMAP, 0.1 eq.) for Wang resin or Fmoc deprotected and then coupled with HATU (4 eq.) and DIEA (8 eq.) for Rink amide resin. Fmoc removal was performed with 20% piperidine in DMF solution at room temperature for 10-12 m. Couplings were performed with a Fmoc amino acid (4 eq.), HATU (4 eq.) and DIEA (8 eq.) or a dipeptide (2 eq.), HATU (2 eq.) and DIEA (4 eq.) or DIC (6 eq.), Oxyma (4 eq.) and DIEA (8 eq.) at room temperature for 60 m. Resin cleavage was performed with 95%TFA/2%H_2O/2%TIPS.

NMR Spectroscopy

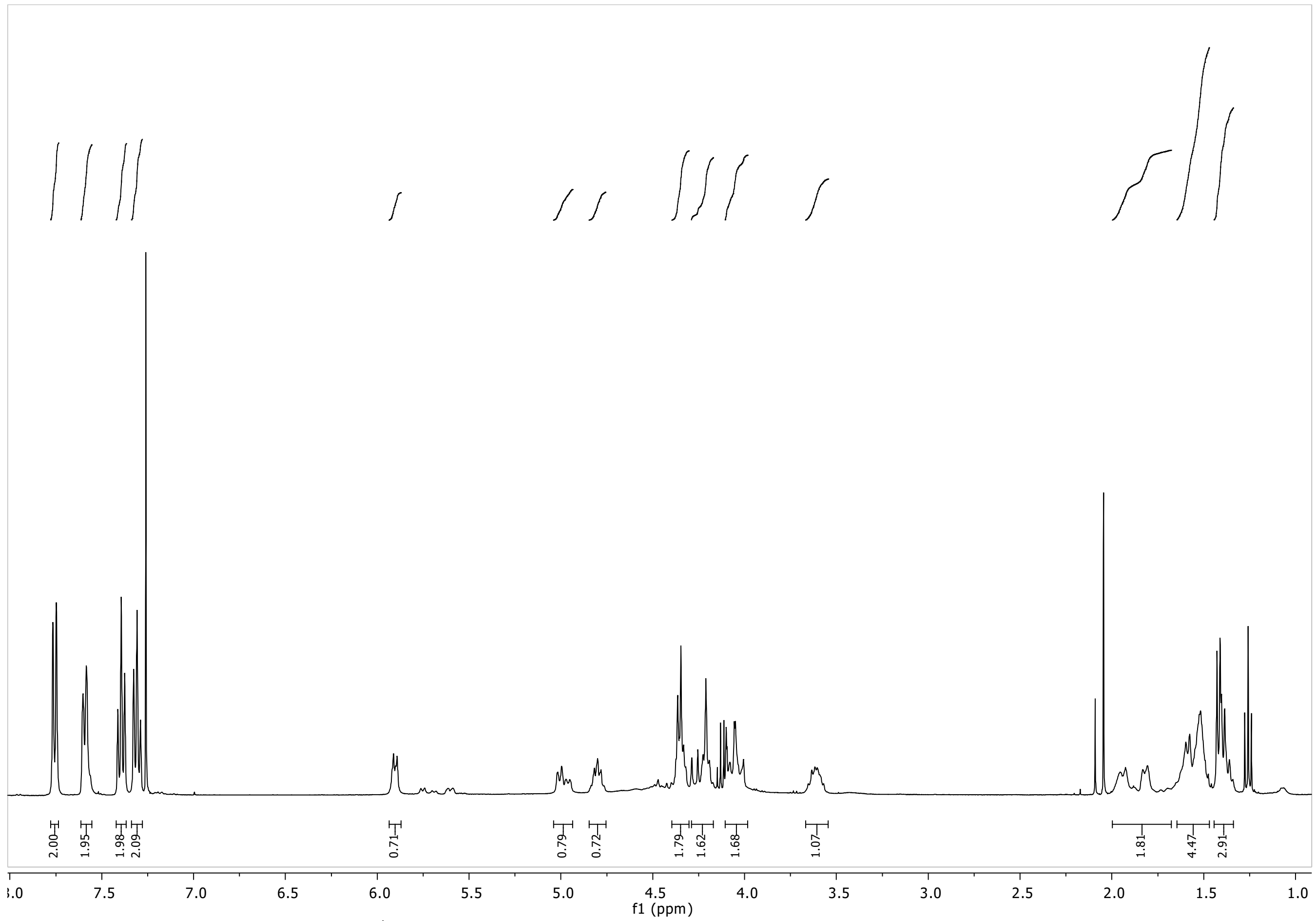

Figure S6: Fmoc-Ala-(Thp)Gly-OH (16) ^{1}HNMR in $CDCl_3$, spectrometer frequency was 400 MHz.

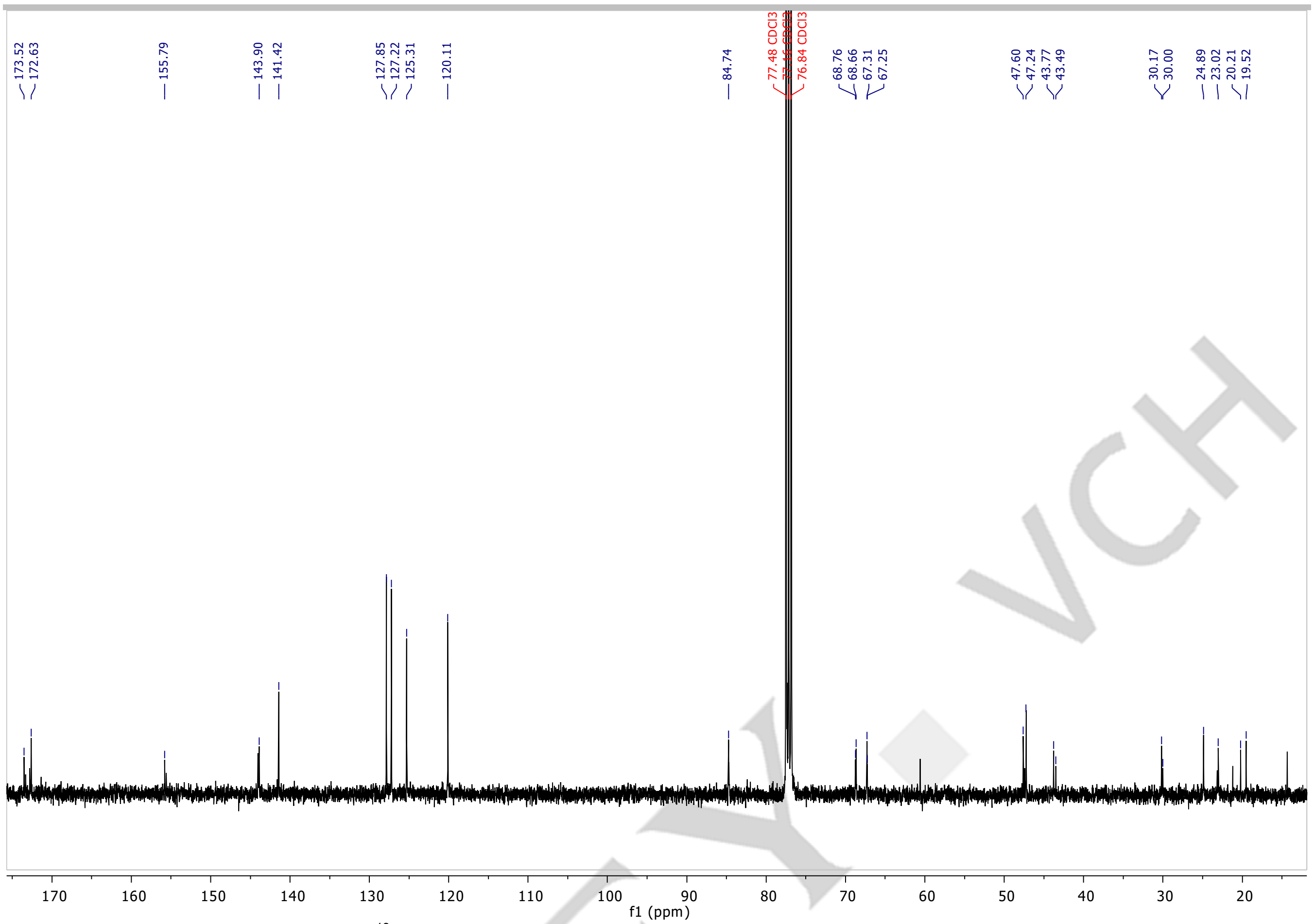

Figure S7: Fmoc-Ala-(Thp)Gly-OH (**16**) ^{13}CNMR in CDCl3, spectrometer frequency was 101 MHz.

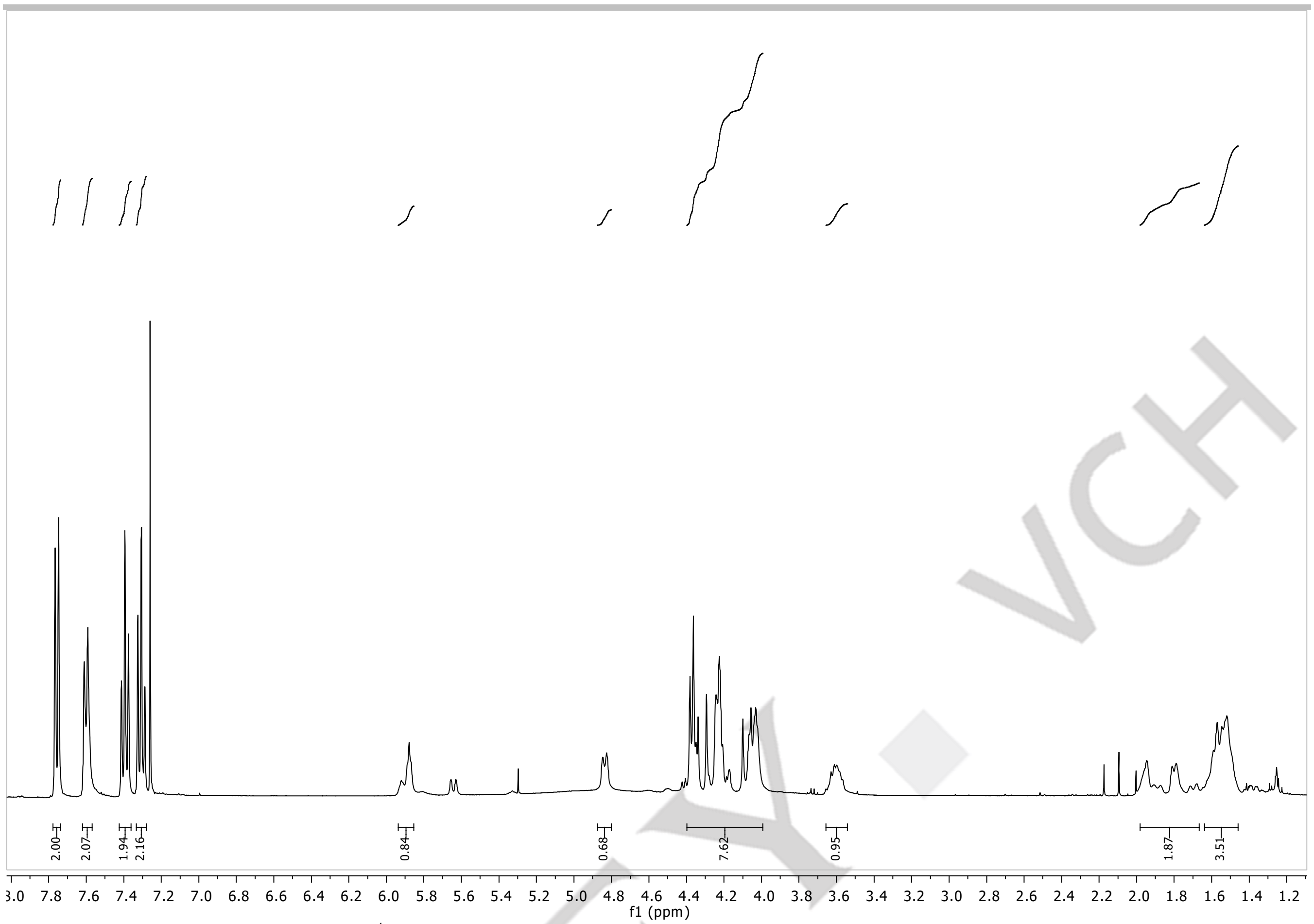

Figure S8: Fmoc-Gly-(Thp)Gly-OH (**14**) ^{1}HNMR in $CDCl_3$, spectrometer frequency was 400 MHz.

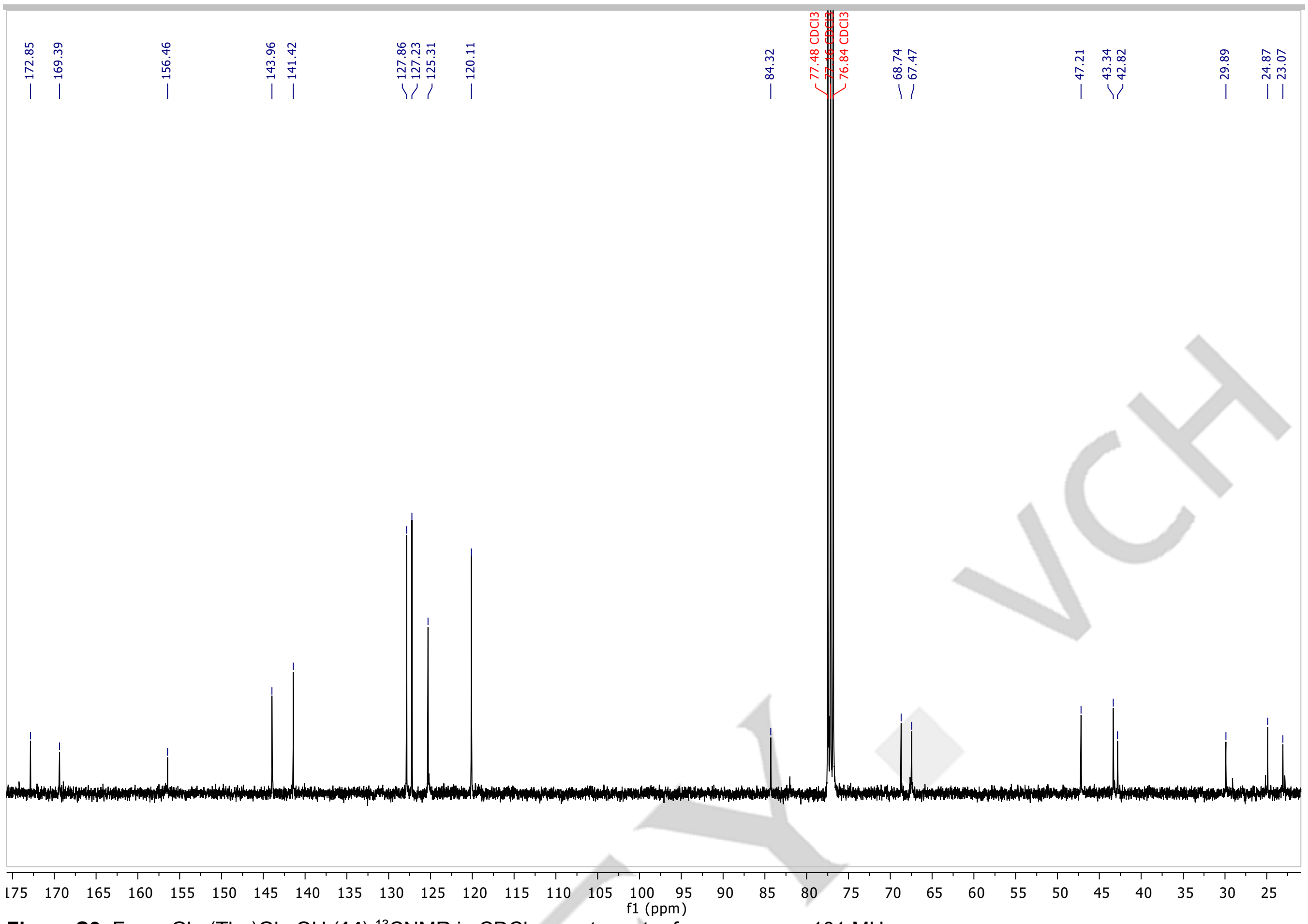

Figure S9: Fmoc-Gly-(Thp)Gly-OH (**14**) ^{13}CNMR in $CDCl_3$, spectrometer frequency was 101 MHz.

WILEY-VCH

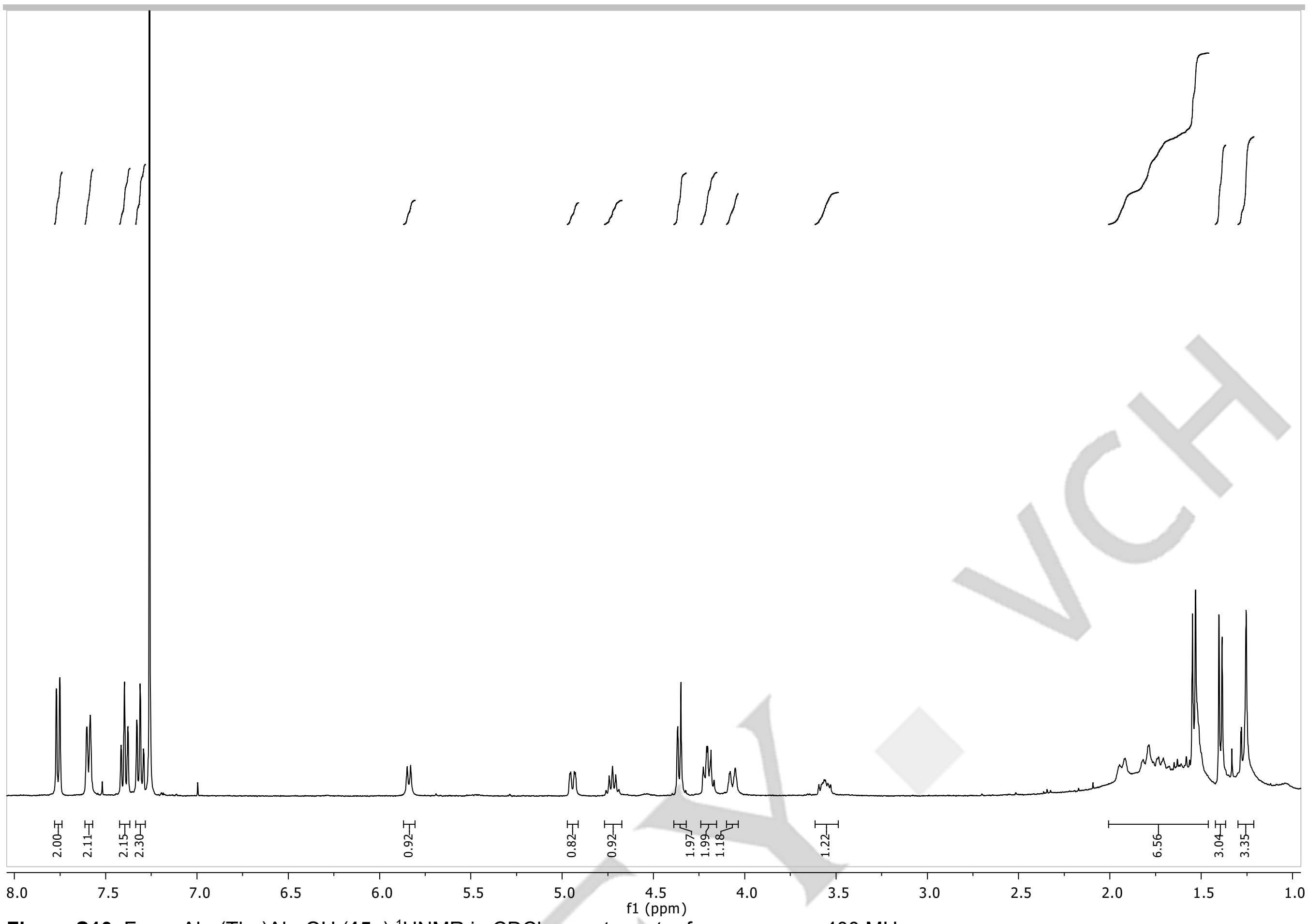

Figure S10: Fmoc-Ala-(Thp)Ala-OH (**15a**) ^{1}HNMR in $CDCl_3$, spectrometer frequency was 400 MHz.

WILEY-VCH

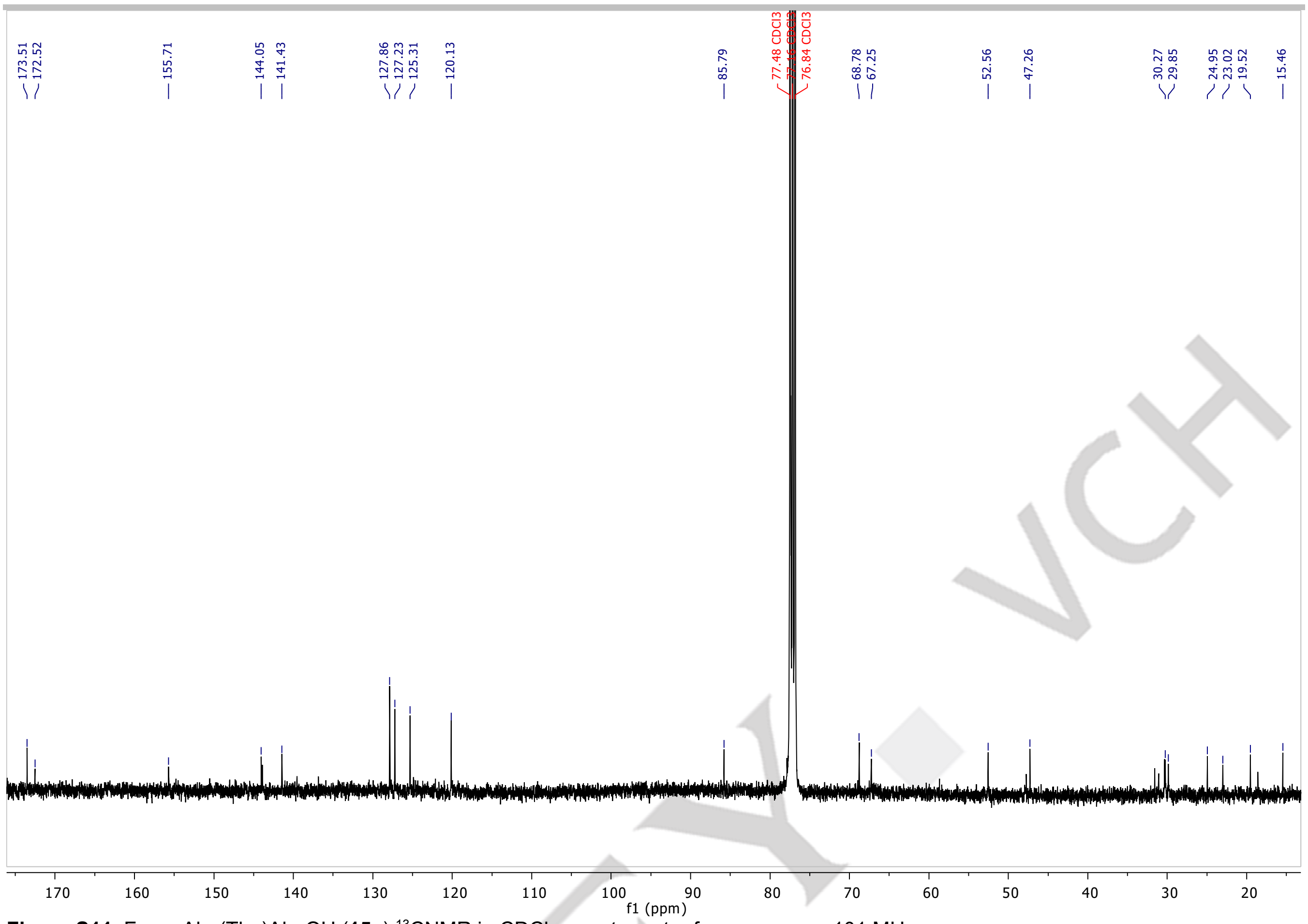

Figure S11: Fmoc-Ala-(Thp)Ala-OH (**15a**) ^{13}CNMR in $CDCl_3$, spectrometer frequency was 101 MHz.

WILEY-VCH

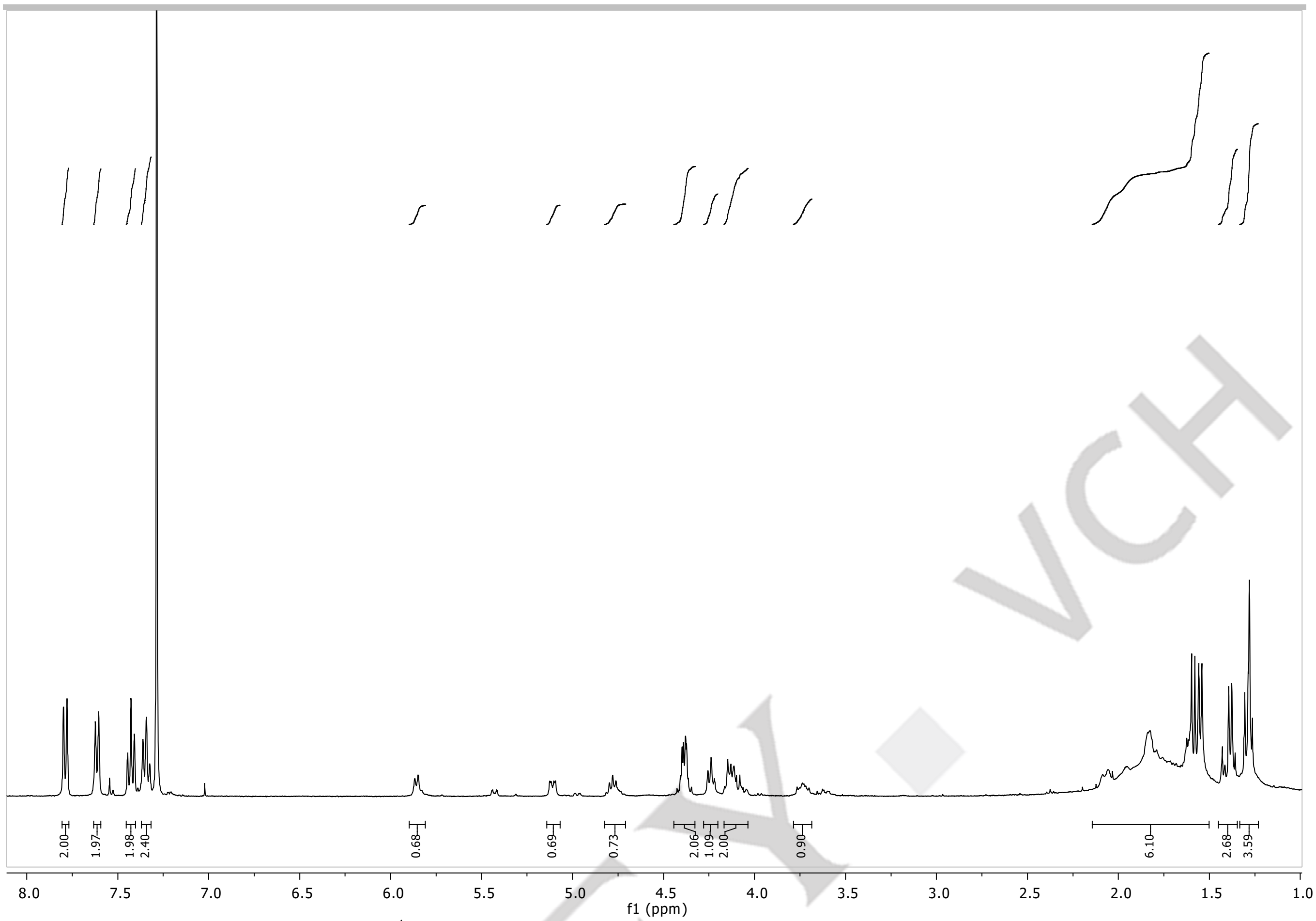

Figure S12: Fmoc-Ala-(Thp)Ala-OH (**15b**) ^{1}HNMR in $CDCl_3$, spectrometer frequency was 400 MHz.

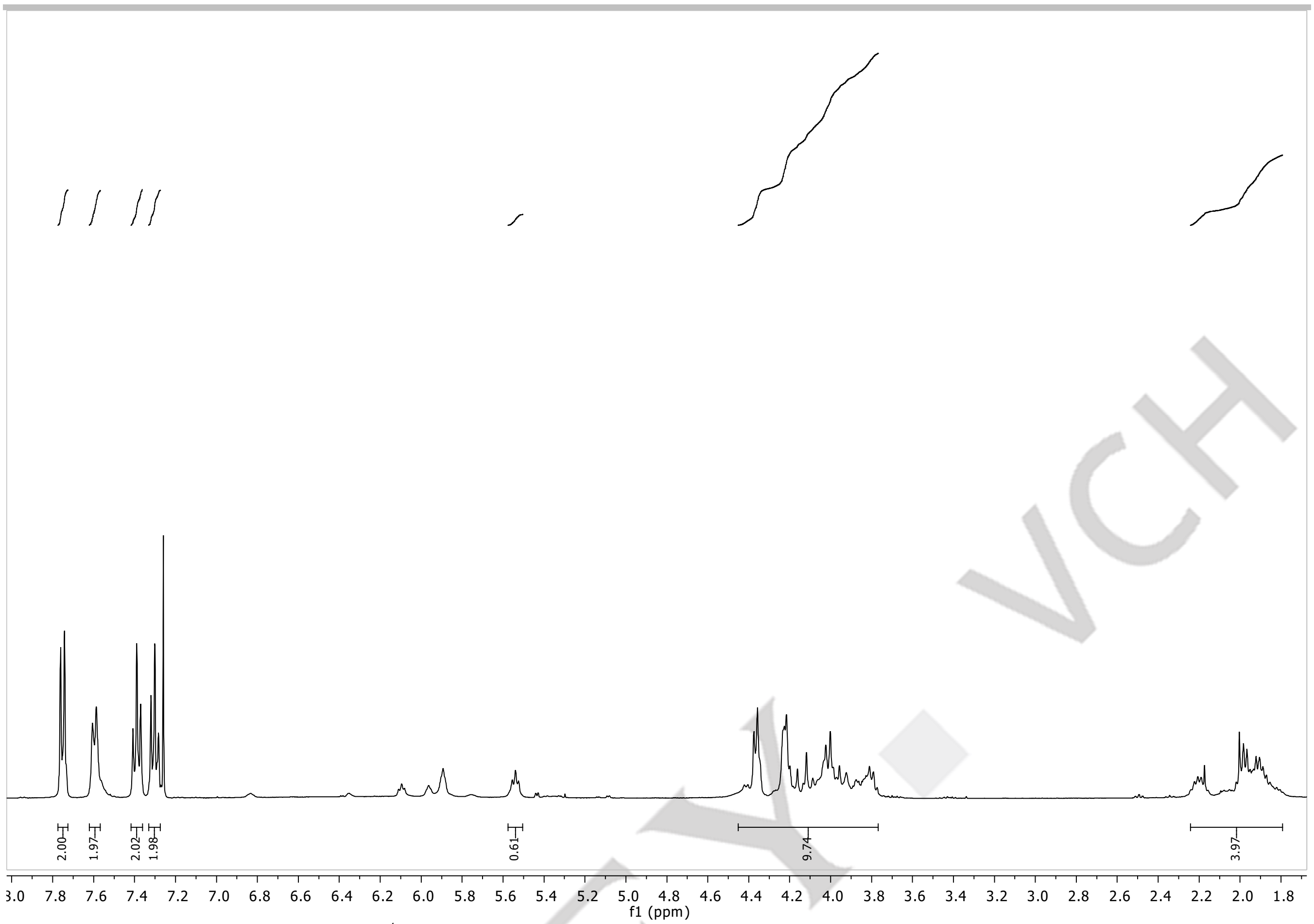

Figure S13: Fmoc-Gly-(Thf)Gly-OH (**17**) ^{1}HNMR in $CDCl_3$, spectrometer frequency was 400 MHz. Appears to degrade to **18** through acidic cleavage of the Thf group in solution (by other **17** or **18** molecules). The triplet at 5.89 ppm suggests the formation of another amide proton (NH) in $CDCl_3$, even when analysed directly after purification. This was followed by precipitation of the impurity if left in solution overnight.

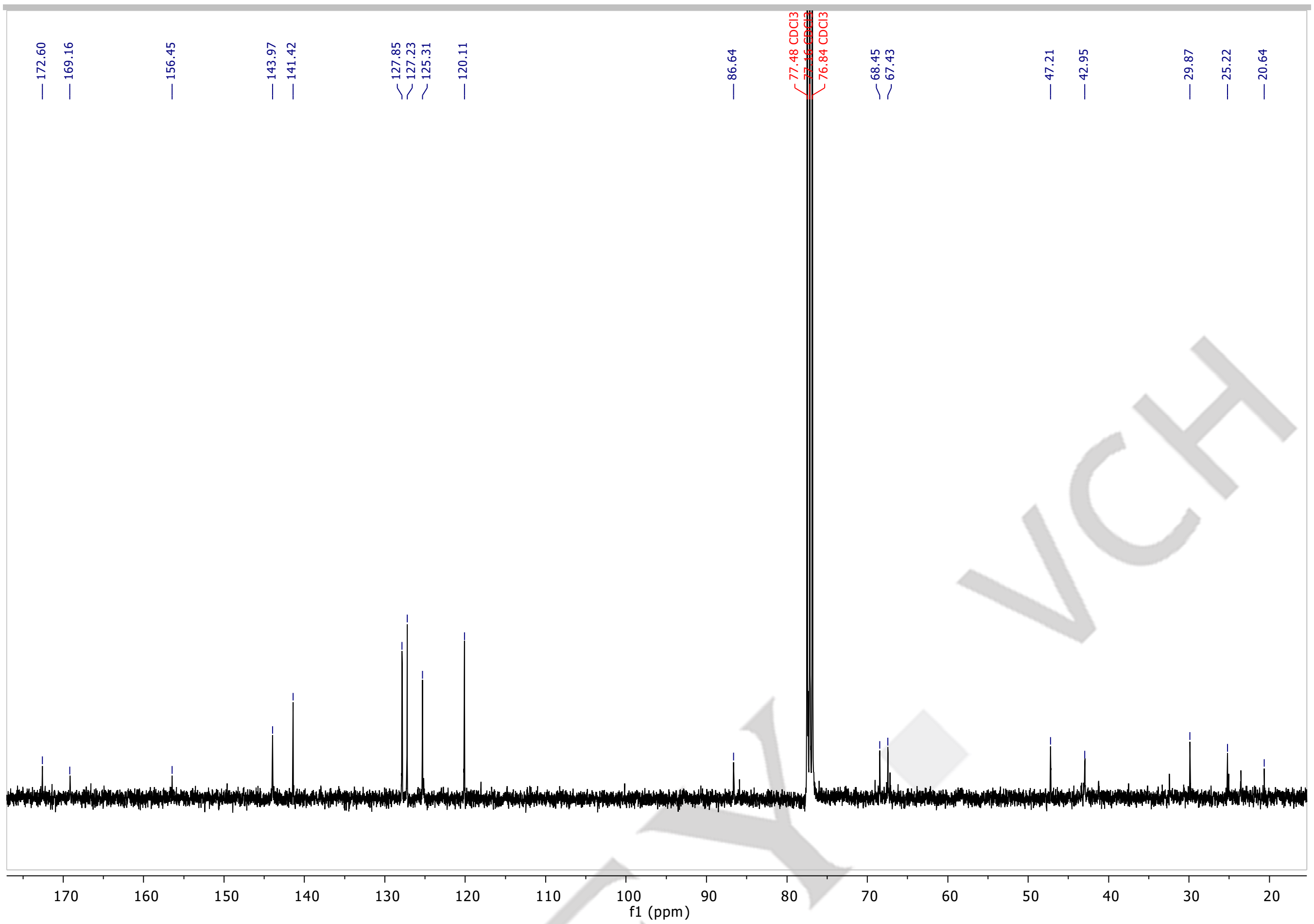

Figure S14: Fmoc-Gly-(Thf)Gly-OH (**17**) ^{13}CNMR in $CDCl_3$, spectrometer frequency was 101 MHz.

Compounds

2.12 Chemical Characterisation

N-Benzylbenzamide (1)

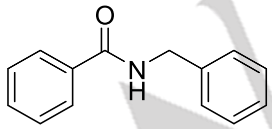

Synthesised via **2.4c** with benzylamine, in 72% yield. Purified by trituration in diethyl ether. ^{1}H NMR (400 MHz, Chloroform-*d*) δ 7.82 – 7.77 (m, 2H, Ar-H), 7.52 – 7.47 (m, 1H, Ar-H), 7.45 – 7.39 (m, 2H, Ar-H), 7.39 – 7.33 (m, 4H, Ar-H), 7.33 – 7.27 (m, 1H, Ar-H), 6.52 (br m, 1H, NH), 4.64 (d, *J* = 5.7 Hz, 2H, CH_2). ^{13}C NMR (101 MHz, $CDCl_3$) δ 167.50, 138.32, 134.51, 131.67, 128.91, 128.71, 128.04, 127.74, 127.09, 44.25. HRMS (ESI): *m/z* calcd for $C_{14}H_{13}NO+H^+$: 212.1075 [*M*+H]$^+$; found: 212.1069; white solid.

N-Dicyclopropylmethylbenzylamine (2)

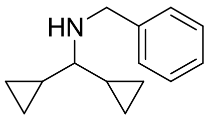

Synthesised in 54% yield. Purification method is detailed in **2.2**. ^{1}H NMR (400 MHz, Chloroform-*d*) δ 7.32 – 7.26 (m, 4H, Ar-H), 7.22 – 7.17 (m, 1H, Ar-H), 3.90 (s, 2H, CH_2), 1.72 (br s, 1H, NH), 1.18 (t, *J* = 8.5 Hz, 1H, CH), 0.90 (dtt, *J* = 8.3, 5.1, 5.1 Hz, 2H, 2 x CH), 0.52 – 0.40 (m, 4H, 2 x CH_2), 0.27 – 0.05 (m, 4H, 2 x CH_2). ^{13}C NMR (101 MHz, $CDCl_3$) δ 140.95, 128.20, 127.81, 126.58, 66.61, 51.89, 16.19, 3.06, 1.77. HRMS (ESI): *m/z* calcd for $C_{14}H_{19}N+H^+$: 202.1596 [*M*+H]$^+$; found: 202.1595; clear oil.

_N_-Dicyclopropylmethyl-_N_-benzylbenzamide (3)

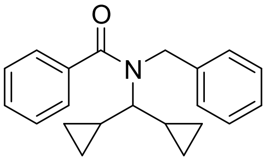

Synthesised via **2.4c** from **2**, in 77% yield. Purified by flash chromatography, ethyl acetate (5 – 12%): hexane over 9 CV. Split peaks due to rotational restriction are reported as X/X where X is the shift in ppm. ^{1}H NMR (400 MHz, Chloroform-*d*) δ 7.49 – 7.18 (m, 10H, Ar-H), 4.91 (s, 1H, ½ CH_2), 4.68 (s, 1H, ½ CH_2), 3.47 (br m, integral = 0.41)/2.68 (t, *J* = 8.7 Hz, integral = 0.53, 1H, CH), 1.01 – 0.82 (m, 2H, 2 x CH), 0.62 – 0.07 (m, 8H, 4 x CH_2). ^{13}C NMR (101 MHz, $CDCl_3$) δ 172.82/172.33, 139.58, 138.98, 137.27, 129.19/129.04, 128.52, 128.30, 127.5/127.27, 126.79/126.47, 67.43/64.15, 50.11/45.64, 14.63, 5.42, 2.83. HRMS (ESI): *m/z* calcd for $C_{21}H_{23}NO+H^+$: 306.1858 [*M*+H]$^+$; found: 306.1854; light yellow oil. Rotational restriction was clearly visible in the ^{1}H NMR.

R/S-_N_-Tetrahydropyran benzylamine (4)

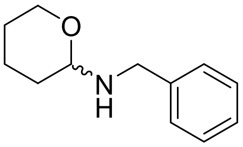

Synthesised via **2.3** from benzylamine and 3,4-dihydro-(2*H*)-pyran in 74% yield. Purified by column chromatography, ethyl acetate (20 – 30%): hexane. ^{1}H NMR (600 MHz, Chloroform-*d*) δ 7.36 – 7.26 (m, 4H, Ar-H), 7.26 – 7.14 (m, 1H, Ar-H), 4.06 (d, *J* = 13.2 Hz, 1H, ½ CH_2), 3.96 (td, *J* = 9.8, 2.8 Hz, 2H, CH_2), 3.83 (d, *J* = 13.2 Hz, 1H, ½ CH_2), 3.45 – 3.39 (m, 1H, CH), 1.86 – 1.74 (m, 2H, CH_2), 1.53 – 1.24 (m, 4H, 2 x CH_2).^{13}C NMR (151 MHz, $CDCl_3$) δ 140.51, 128.32, 128.26, 126.82, 87.43, 66.48, 49.02, 32.55, 25.78, 23.34. HRMS (ESI): *m/z* calcd for $C_{12}H_{17}NO+H^+$: 192.1388 [*M*+H]$^+$; found: 192.1383; clear oil.

R/S-_N_-Tetrahydropyran-_N_-benzylbenzamide (5)

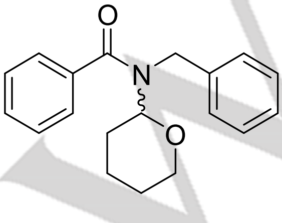

Synthesised via **2.4c** from **4** and benzoyl chloride, in 79% yield. Purified by flash chromatography, ethyl acetate (0 – 5%): hexane over 7 CV. ^{1}H NMR (600 MHz, Chloroform-*d*) δ 7.63 – 7.27 (m, 9H, Ar-H), 7.26 – 7.15 (m, 1H, Ar-H), 5.11 (br s, 1H, ½ CH_2), 4.79 (br s, 1H, ½ CH_2), 4.50 (d, *J* = 15.9 Hz, 1H, ½ CH_2), 4.03 (d, *J* = 12.0 Hz, 1H, ½ CH_2), 3.32 (br m, 1H, CH), 1.82 – 1.54 (m, 2H, CH_2), 1.53 – 1.22 (m, 4H, 2 x CH_2). ^{13}C NMR (151 MHz, $CDCl_3$) δ 172.74, 139.29, 136.29, 130.05, 129.92, 128.36, 128.18, 127.01, 126.69, 87.06, 67.95, 45.42, 30.63, 24.91, 23.34. HRMS (ESI): *m/z* calcd for $C_{19}H_{21}NO_2+H^+$: 296.1651 [*M*+H]$^+$; found: 296.1644; clear oil. Rotational restriction was clearly visible in the ^{1}H NMR.

H-(R/S-Thp)Gly-OBn (6)

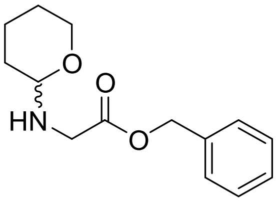

Synthesised via **2.3** from glycine benzyl ester hydrochloride and 3,4-dihydro-(2*H*)-pyran in 76% yield. No purification required. ^{1}H NMR (400 MHz, Chloroform-*d*) δ 7.35 – 7.26 (m, 5H, Ar-H), 5.14 (s, 2H, CH_2), 3.93 – 3.85 (m, 2H, CH_2), 3.58 (dd, *J* = 17.4 Hz, 17.4 Hz, 2H, CH_2), 3.40 – 3.32 (m, 1H, CH), 2.19 (br s, 1H, NH), 1.84 – 1.74 (m, 2H, CH_2), 1.54 – 1.24 (m, 4H, 2 x CH_2). ^{13}C NMR (101 MHz, $CDCl_3$) δ 172.44, 135.73, 128.47, 128.22, 128.20, 87.52, 66.38, 66.29, 46.79, 32.04, 25.50, 23.03. HRMS (ESI): *m/z* calcd for $C_{14}H_{19}NO_3+H^+$: 250.1443 [*M*+H]$^+$; found: 250.1436; clear oil.

H-(R/S-Thf)Gly-OBn (7)

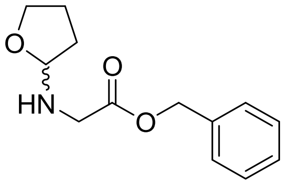

Synthesised via **2.3** from glycine benzyl ester hydrochloride and 2,3-dihydrofuran in 62% yield. No purification required.^{1}H NMR (400 MHz, Chloroform-*d*) δ 7.36 – 7.29 (m, 5H, Ar-H), 5.15 – 5.12 (m, 2H, CH_2), 4.77 – 4.70 (m, 1H, CH), 3.80 – 3.66 (m, 2H, CH_2), 3.53 (dd, *J* = 17.3, 5.0 Hz, 2H, CH_2), 3.44 – 3.41 (m, 1H, NH), 2.14 – 1.53 (m, 4H, 2 x CH_2). ^{13}C NMR (101 MHz, $CDCl_3$) δ 172.60, 135.73, 128.61, 128.55, 128.32, 90.46, 66.54, 66.21, 47.17, 31.85, 25.02. HRMS (ESI): *m/z* calcd for $C_{13}H_{17}NO_3+H^+$: 236.1287 [*M*+H]$^+$; found: 236.1279; clear oil.

H-(R/S-Thp)-L-Ala-OBn (8)

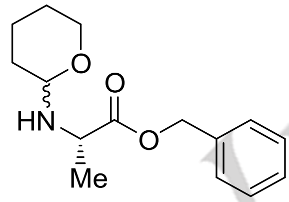

Synthesised via **2.3** from alanine benzyl ester hydrochloride and 3,4-dihydro-(2*H*)-pyran in 87% yield. No purification required. ^{1}H NMR (400 MHz, Chloroform-*d*) δ 7.26 – 7.15 (m, 5H, Ar-H), 5.09 – 4.96 (m, 2H, CH_2), 3.86 – 3.67 (m, 2H, CH_2), 3.49 (q, *J* = 7.0 Hz, 1H, CH), 3.27 – 3.16 (m, 1H, CH), 2.40 (br s, 1H, NH), 1.77 – 1.58 (m, 2H, CH_2), 1.43 – 1.25 (m, 4H, 2 x CH_2), 1.23 (minor peak, d, *J* = 7.1 Hz, integration = 1.26, 1H)/1.18 (major peak, d, *J* = 7.0, 2.7 Hz, integral = 2.33, 2H, CH_3). Diastereomers reported as X/X where X is the shift in ppm. ^{13}C NMR (101 MHz, $CDCl_3$) δ 175.70/174.87, 135.74/135.68, 128.08/128.02, 127.88, 127.72/127.65, 86.75/86.34, 65.94/65.84, 65.82/65.68, 52.90/52.03, 31.86/31.67, 25.11/25.07, 22.86/22.60, 18.99/18.70. HRMS (ESI): *m/z* calcd for $C_{15}H_{21}NO_3+H^+$: 264.1600 [*M*+H]$^+$; found: 264.1597; clear oil.

Fmoc-Gly-(R/S-Thp)Gly-OBn (9)

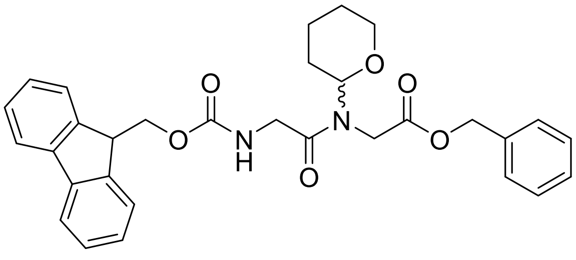

Synthesised from **6** and Fmoc-Gly-OH via **2.4d** in 80% yield, the crude mixture was not washed with HCl aq. Purified by flash chromatography, ethyl acetate (5 - 20%): hexane over 7 CV, held at ethyl acetate (20%): hexane for 1 CV, then ethyl acetate (20 - 100%): hexane over 10 CV.^{1}H NMR (400 MHz, Chloroform-*d*) δ 7.77 (d, *J* = 7.5 Hz, 2H, Ar-H), 7.61 (d, *J* = 7.5 Hz, 2H, Ar-H), 7.44 – 7.29 (m, 9H, Ar-H), 5.82 (t, *J* = 4.2 Hz, 1H, NH), 5.20 – 5.16 (m, 2H, CH_2), 4.80 (t, *J* = 9.5 Hz, 1H, CH), 4.43 – 4.33 (m, 2H, CH_2), 4.31 – 3.89 (m, 6H, 3 x CH_2), 3.63 – 3.52 (m, 1H, CH), 2.02 – 1.69 (m, 2H, CH_2), 1.57 – 1.44 (m, 4H, 2 x CH_2). ^{13}C NMR (101 MHz, $CDCl_3$) δ 169.31, 168.83, 156.27, 144.02, 141.42, 135.54, 128.74, 128.54, 128.41, 127.83, 127.22, 125.32, 120.10, 84.28, 68.62, 67.35, 67.17, 47.25, 43.36, 42.83, 29.94, 24.91, 23.17. HRMS (ESI): *m/z* calcd for $C_{31}H_{32}N_2O_6$+H^+: 529.2339 [*M*+H]$^+$; found: 529.2334; white, brittle powder.

Fmoc-L-Ala-(R/S-Thp)-L-Ala-OBn (10a)

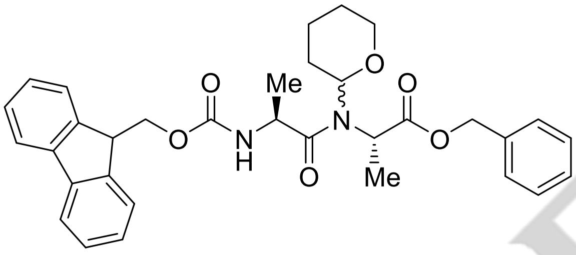

Synthesised from **8** and Fmoc-Ala-OH via **2.4b** in 16% yield, the crude mixture was not washed with HCl aq. Purified by flash chromatography, ethyl acetate (5 - 25%): hexane over 7 CV, held at ethyl acetate (25%): hexane for 6 CV, then ethyl acetate (25 - 40%): hexane over 8 CV, then held at ethyl acetate (40%): hexane for 5 CV. ^1H NMR (400 MHz, Chloroform-*d*) δ 7.70 – 7.66 (m, 2H, Ar-H), 7.55 – 7.51 (m, 2H, Ar-H), 7.34 – 7.28 (m, 3H, Ar-H), 7.25 – 7.14 (m, 6H, Ar-H), 5.85 (d, *J* = 7.9 Hz, 1H, NH), 5.14 – 4.99 (m, 2H, CH_2), 4.92 – 4.86 (m, 1H, CH), 4.70 – 4.62 (m, 1H, CH), 4.29 (m, 2H, CH_2), 4.15 – 4.09 (m, 2H, CH_2), 3.99 – 3.92 (m, 1H, CH), 3.56 – 3.38 (m, 1H, CH), 1.84 – 1.53 (m, 4H, 2 x CH_2), 1.49 (d, *J* = 6.8 Hz, 3H, CH_3), 1.46 – 1.36 (m, 2H, CH_2), 1.31 (d, *J* = 6.8 Hz, 3H, CH_3). ^{13}C NMR (101 MHz, $CDCl_3$) δ 171.29, 170.88, 155.57, 144.01, 143.89, 141.36, 136.01, 128.53, 128.07, 127.79, 127.15, 125.20, 120.05, 85.63, 68.71, 67.07, 66.86, 51.86, 47.51, 47.22, 30.38, 24.96, 23.01, 19.47, 15.48. HRMS (ESI): *m/z* calcd for $C_{33}H_{36}N_2O_6$+H^+: 557.2652 [*M*+H]$^+$; found: 557.2640; white, brittle powder.

Fmoc-L-Ala-(R/S-Thp)-L-Ala-OBn (10b)

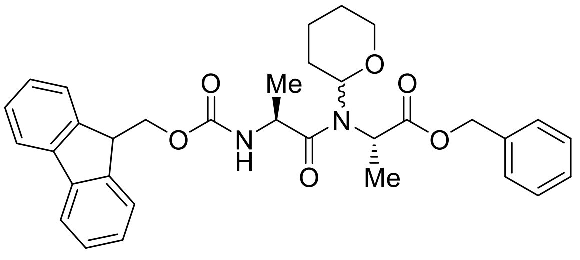

Synthesised from **8** and Fmoc-Ala-OH via **2.4b** in 16% yield, the crude mixture was not washed with HCl aq. Purified by flash chromatography, ethyl acetate (5 - 25%): hexane over 7 CV, held at ethyl acetate (25%): hexane for 6 CV, then ethyl acetate (25 - 40%): hexane over 8 CV, then held at ethyl acetate (40%): hexane for 5 CV. ^{1}H NMR (400 MHz, Chloroform-*d*) δ 7.79 (d, *J* = 7.5 Hz, 2H, Ar-H), 7.64 – 7.58 (m, 2H, Ar-H), 7.45 – 7.40 (m, 2H, Ar-H), 7.39 – 7.26 (m, 7H, Ar-H), 5.72 (d, *J* = 7.7 Hz, 1H, NH), 5.29 – 5.11 (m, 2H, CH_2), 4.89 – 4.83 (m, 1H, CH), 4.82 – 4.76 (m, 1H, CH), 4.42 – 4.33 (m, 3H, CH_2 + CH), 4.25 – 4.03 (m, 2H, CH_2), 3.63 – 3.54 (m, 1H, CH), 2.04 – 1.69 (m, 4H, 2 x CH_2), 1.66 – 1.49 (m, 5H, CH_3 + CH_2), 1.34 (d, *J* = 6.8 Hz, 3H, CH_3). ^{13}C NMR (101 MHz, $CDCl_3$) δ 171.90, 171.04, 155.46, 144.11, 143.93, 141.40, 136.09, 128.51, 128.07, 127.81, 127.18, 125.30, 120.07, 85.15, 68.79, 67.04, 66.84, 52.02, 47.77, 47.28, 30.87, 24.89, 23.52, 20.12, 16.03. HRMS (ESI): *m/z* calcd for $C_{33}H_{36}N_2O_6+H^+$: 557.2652 [*M*+H]$^+$; found: 557.2641; white, brittle powder.

Fmoc-L-Ala-(R/S-Thp)Gly-OBn (11)

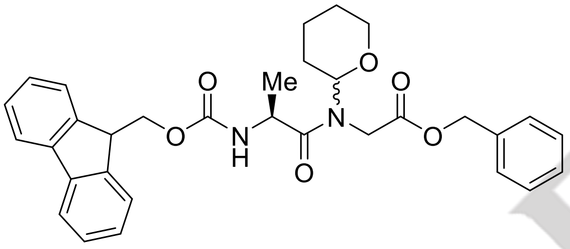

Synthesised from **6** and Fmoc-Ala-OH via **2.4b** in 26% yield, the crude mixture was not washed with HCl aq. Purified by column chromatography, ethyl acetate (10 - 30%): hexane. ^{1}H NMR (400 MHz, Chloroform-*d*) δ 7.77 (d, *J* = 7.5 Hz, 2H, Ar-H), 7.61 (d, *J* = 7.1 Hz, 2H, Ar-H), 7.44 – 7.28 (m, 9H, Ar-H), 5.89 (m, 1H, NH), 5.17 (s, 2H, CH_2), 5.01 – 4.85 (m, 1H, CH), 4.85 – 4.75 (m, 1H, CH), 4.39 – 4.34 (m, 2H, CH_2), 4.31 – 4.19 (m, 2H, CH_2), 4.09 – 3.93 (m, 2H, CH_2), 3.64 – 3.52 (m, 1H, CH), 1.98 – 1.75 (m, 2H, CH_2), 1.66 – 1.45 (m, 4H, 2 x CH_2), 1.40 (dd, *J* = 12.8, 6.8 Hz, 3H, CH_3). Diastereomers reported as X/X where X is the shift in ppm. ^{13}C NMR (101 MHz, $CDCl_3$) δ 172.84/172.80, 169.25/169.20, 155.65/155.50, 144.07/143.93, 141.39, 135.59/135.53, 128.71/128.68, 128.51/128.46, 128.38/128.35, 127.81, 127.19, 125.29, 120.07, 84.67/84.61, 68.61/68.51, 67.18/67.11, 67.08/67.04, 47.40/47.26, 43.58/43.33, 30.23/30.01, 24.90, 23.24/23.09, 20.30, 19.70. HRMS (ESI): *m/z* calcd for $C_{32}H_{34}N_2O_6+H^+$: 543.2495 [*M*+H]$^+$; found: 543.2481; white, brittle powder.

Fmoc-Gly-(R/S-Thf)Gly-OBn (12)

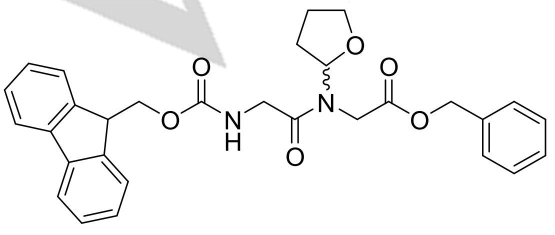

Synthesised from **7** and Fmoc-Gly-OH via **2.4b** in 11% yield, the crude mixture was not washed with HCl. Purified by flash chromatography, ethyl acetate (15 - 40%): hexane over 8 CV, ethyl acetate (40 - 70%): hexane over 1 CV, and then held at ethyl acetate (70%): hexane for 2 CV. ^{1}H NMR (400 MHz, Chloroform-*d*) δ 7.77 (d, *J* = 7.5 Hz, 2H, Ar-H), 7.62 (d, *J* = 7.5 Hz, 2H, Ar-H), 7.44 – 7.29 (m, 9H, Ar-H), 5.52 (t, *J* = 6.2 Hz, 1H, NH), 5.19 (s, 2H, CH_2),

4.38 (d, *J* = 5.5 Hz, 2H, CH_2), 4.30 – 3.87 (m, 7H, CH + 3 x CH_2), 3.82 – 3.73 (m, 1H, CH), 2.24 – 1.72 (m, 4H, 2 x CH_2). ^{13}C NMR (101 MHz, $CDCl_3$) δ 169.27, 168.58, 156.23, 143.98, 141.37, 135.47, 128.71, 128.53, 128.38, 127.79, 127.18, 125.28, 120.05, 86.50, 68.24, 67.29, 67.20, 47.20, 42.99, 42.93, 29.84, 25.13. HRMS (ESI): *m/z* calcd for $C_{30}H_{30}N_2O_6+H^+$: 515.2182 [*M*+H]$^+$; found: 515.2188; white, brittle powder.

Fmoc-Gly-Gly-OtBu (13)

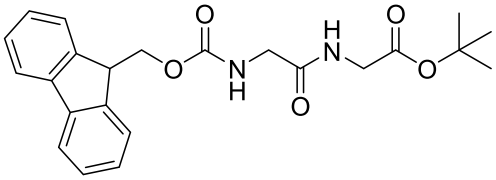

Synthesised from glycine *t*-butyl ester hydrochloride and Fmoc-Gly-OH via **2.4a** in 70% yield. Purified by recrystallisation in ethyl acetate: hexane. ^{1}H NMR (400 MHz, Chloroform-*d*) δ 7.77 (d, *J* = 7.6, 2H, Ar-H), 7.58 (d, *J* = 7.5 Hz, 2H, Ar-H), 7.38 (app. td, *J* = 7.4, 1.2 Hz, 2H, Ar-H), 7.29 (app. td, *J* = 7.4, 1.2 Hz, 2H, Ar-H), 6.64 (br m, 1H, NH), 5.72 (t, *J* = 5.8 Hz, 1H, NH), 4.42 (d, *J* = 7.0 Hz, 2H, CH_2), 4.22 (t, *J* = 6.9 Hz, 1H, CH), 3.93 (t, *J* = 5.6 Hz, 4H, 2 x CH_2), 1.46 (s, 9H, 3 x CH_3). ^{13}C NMR (101 MHz, $CDCl_3$) δ 169.25, 168.91, 156.75, 143.84, 141.38, 127.81, 127.18, 125.16, 120.08, 82.59, 67.29, 47.19, 44.45, 42.02, 28.11. HRMS (ESI): *m/z* calcd for $C_{23}H_{26}N_2O_5+H^+$: 411.1920 [*M*+H]$^+$; found: 411.1918; white solid.

Fmoc-Gly-(R/S-Thp)Gly-OH (14)

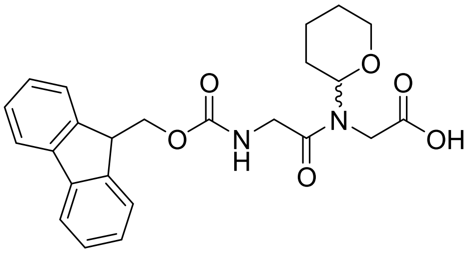

Synthesised via **2.5** from **9** in 74% yield. Purified by flash chromatography, CH_2Cl_2 : methanol (0 – 9%) + acetic acid (0.1 %v/v) over 10 CV. ^{1}H NMR (400 MHz, Chloroform-*d*) δ 7.76 (d, *J* = 7.5 Hz, 2H, Ar-H), 7.60 (d, *J* = 7.3 Hz, 2H, Ar-H), 7.39 (app. td, *J* = 7.4 Hz, 2H, Ar-H), 7.31 (app. td, *J* = 7.4, 1.2 Hz, 2H, Ar-H), 5.90 (t, *J* = 4.5 Hz, 1H, NH), 4.88 – 4.80 (m, 1H, CH), 4.40 – 3.99 (m, 8H, 4 x CH_2), 3.66 – 3.54 (m, 1H, CH), 1.98 – 1.67 (m, 2H, CH_2), 1.64 – 1.46 (m, 4H, 2 x CH_2). ^{13}C NMR (101 MHz, $CDCl_3$) δ 172.85, 169.39, 156.46, 143.96, 141.42, 127.86, 127.23, 125.31, 120.11, 84.32, 68.74, 67.47, 47.21, 43.34, 42.82, 29.89, 24.87, 23.07. HRMS (ESI): *m/z* calcd for $C_{24}H_{26}N_2O_6+H^+$: 439.1869 [*M*+H]$^+$; found: 439.1857; white lyophilised powder or clear, sticky gum.

Fmoc-L-Ala-(R/S-Thp)-L-Ala-OH (15a)

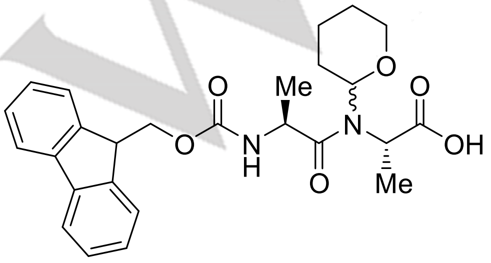

Synthesised via **2.5** from **10** in 40% yield. Purified by flash chromatography, CH_2Cl_2 : methanol (0 – 9%) + acetic acid (0.1 %v/v) over 10 CV. ^{1}H NMR (400 MHz, Chloroform-*d*) δ 7.76 (d, *J* = 7.5 Hz, 2H, Ar-H), 7.59 (d, *J* = 7.5 Hz, 2H, Ar-H), 7.40 (app. td, *J* = 7.5, 1.2 Hz, 2H, Ar-H), 7.31 (app. td, *J* = 7.5, 1.2 Hz, 2H, Ar-H), 5.84 (d, *J* = 7.7 Hz, 1H, NH), 4.97 – 4.91 (m, 1H, CH), 4.72 (app. p, *J* = 7.0 Hz, 1H, CH), 4.39 – 4.32 (m, 2H, CH_2), 4.24 – 4.15 (m, 2H, CH_2), 4.10 – 4.04 (m, 1H, CH), 3.62 – 3.49 (m, 1H, CH), 2.01 – 1.46 (m, 6H, 3 x CH_2), 1.39 (d, *J* = 6.8 Hz, 3H,

CH_3), 1.30 – 1.21 (m, 3H, CH_3). ^{13}C NMR (101 MHz, $CDCl_3$) δ 173.51, 172.52, 155.71, 144.05, 141.43, 127.86, 127.23, 125.31, 120.13, 85.79, 68.78, 67.25, 52.56, 47.26, 30.27, 29.85, 24.95, 23.02, 19.52, 15.46. HRMS (ESI): *m/z* calcd for $C_{26}H_{30}N_2O_6+H^+$: 467.2182 [*M*+H]$^+$; found: 467.2174; white lyophilised powder or clear, sticky gum.

Fmoc-L-Ala-(R/S-Thp)-L-Ala-OH (15b)

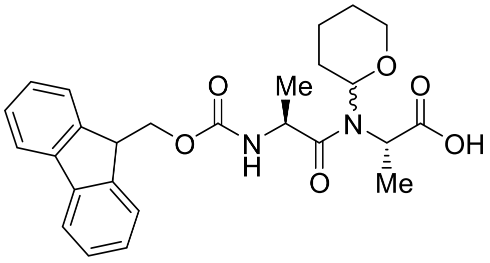

Synthesised via **2.5** from **10** in 62% yield. Purified by flash chromatography, CH_2Cl_2 : methanol (0 – 9%) + acetic acid (0.1 %v/v) over 10 CV. ^{1}H NMR (400 MHz, Chloroform-*d*) δ 7.79 (d, *J* = 7.5 Hz, 2H, Ar-H), 7.61 (d, *J* = 7.5 Hz, 2H, Ar-H), 7.45 – 7.40 (m, 2H, Ar-H), 7.37 – 7.32 (m, 2H, Ar-H), 5.86 (d, *J* = 7.6 Hz, 1H, NH), 5.14 – 5.07 (m, 1H, CH), 4.82 – 4.71 (m, 1H, CH), 4.44 – 4.33 (m, 2H, CH_2), 4.24 (t, *J* = 7.3 Hz, 1H, CH), 4.17 – 4.04 (m, 2H, CH_2), 3.79 – 3.69 (m, 1H, CH), 2.14 – 1.50 (m, 6H, 3 x CH_2), 1.45 – 1.35 (m, 3H, CH_3), 1.33 – 1.23 (m, 3H, CH_3). ^{13}C NMR (101 MHz, CDCl3) δ 172.33, 167.85, 155.56, 143.93, 141.28, 127.71, 127.11, 125.24, 119.96, 84.99, 68.97, 68.59, 64.53, 52.04, 47.48, 47.09, 30.20, 25.26, 20.75, 15.24. HRMS (ESI): *m/z* calcd for $C_{26}H_{30}N_2O_6+H^+$: 467.2182 [*M*+H]$^+$; found: 467.2177; white lyophilised powder or clear, sticky gum.

Fmoc-L-Ala-(R/S-Thp)Gly-OBn (16)

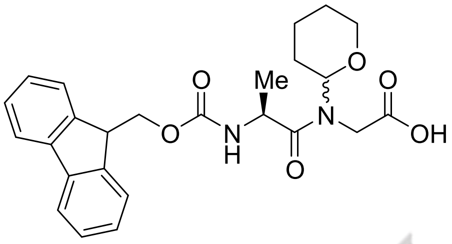

Synthesised via **2.5** from **11** in 81% yield. Purified by column chromatography, CH_2Cl_2 : methanol (0 – 3%) + acetic acid (0.1 %v/v). ^{1}H NMR (400 MHz, Chloroform-*d*) δ 7.76 (d, *J* = 7.5 Hz, 2H, Ar-H), 7.59 (d, *J* = 7.6 Hz, 2H, Ar-H), 7.39 (app. td, *J* = 7.3, 1.2 Hz, 2H, Ar-H), 7.31 (app. td, *J* = 7.3, 1.2 Hz, 2H, Ar-H), 5.94 – 5.87 (m, 1H, NH), 5.04 – 4.94 (m, 1H, CH), 4.85 – 4.76 (m, 1H, CH), 4.40 – 4.30 (m, 2H, CH_2), 4.29 – 4.17 (m, 2H, CH_2), 4.11 – 3.98 (m, 2H, CH_2), 3.67 – 3.54 (m, 1H, CH), 2.00 – 1.68 (m, 2H, CH_2), 1.65 – 1.47 (m, 4H, 2 x CH_2), 1.44 – 1.34 (m, 3H, CH_3). Diastereomers reported as X/X where X is the shift in ppm. ^{13}C NMR (101 MHz, $CDCl_3$) δ 173.52, 172.63, 155.79, 143.90, 141.42, 127.85, 127.22, 125.31, 120.11, 84.74, 68.76/68.66, 67.31/67.25, 47.60/47.24, 43.77/43.49, 30.17/30.00, 24.89, 23.02, 20.21, 19.52. HRMS (ESI): *m/z* calcd for $C_{25}H_{28}N_2O_6+H^+$: 453.2026 [*M*+H]$^+$; found: 453.2020; white lyophilised foam or clear, sticky gum.

WILEY-VCH

Fmoc-Gly-(R/S-Thf)Gly-OH (17)

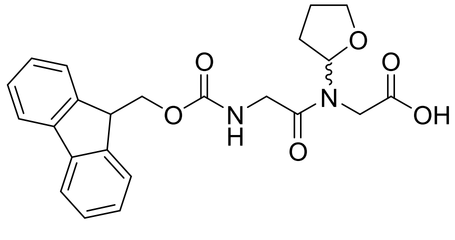

Synthesised via **2.5** from **12** in 73% yield. Purified by flash chromatography, CH_2Cl_2 : methanol (0 – 9%) + acetic acid (0.1 %v/v) over 10 CV. ^{1}H NMR (400 MHz, Chloroform-*d*) δ 7.75 (d, *J* = 7.5 Hz, 2H, Ar-H), 7.60 (d, *J* = 7.5 Hz, 2H, Ar-H), 7.39 (app. td, *J* = 7.5, 1.2 Hz, 2H, Ar-H), 7.30 (app. td, *J* = 7.5, 1.2 Hz, 2H, Ar-H), 5.54 (t, *J* = 6.4 Hz, 1H, NH), 4.45 – 3.77 (m, 10H, 2 x CH + 4 x CH_2), 2.24 – 1.79 (m, 4H, 2 x CH_2). ^{13}C NMR (101 MHz, $CDCl_3$) δ 172.60, 169.16, 156.45, 143.97, 141.42, 127.85, 127.23, 125.31, 120.11, 86.64, 68.45, 67.43, 47.21, 42.95, 29.87, 25.22, 20.64. HRMS (ESI): *m/z* calcd for $C_{23}H_{24}N_2O_6+H^+$: 425.1712 [*M*+H]$^+$; found: 425.1704; white lyophilised powder or clear, sticky gum.

Fmoc-Gly-Gly-OH (18)

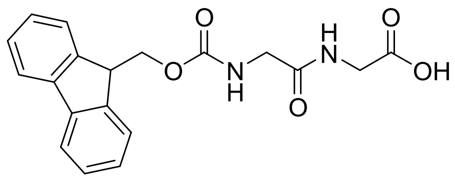

Synthesised via **2.6** from **13** in 68% yield. ^{1}H NMR (400 MHz, DMSO-*d6*) δ 12.53 (br s, 1H, CO_2H), 8.14 (t, *J* = 5.9 Hz, 1H, NH), 7.89 (d, *J* = 7.5 Hz, 2H, Ar-H), 7.72 (d, *J* = 7.5 Hz, 2H, Ar-H), 7.58 (t, *J* = 6.2 Hz, 1H, NH), 7.42 (app. td, *J* = 7.5, 1.2 Hz, 2H, Ar-H), 7.33 (app. td, *J* = 7.5, 1.2 Hz, 2H, Ar-H), 4.29 (d, *J* = 6.2 Hz, 2H, CH_2), 4.25 – 4.20 (m, 1H, CH), 3.77 (d, *J* = 5.8 Hz, 2H, CH_2), 3.65 (d, *J* = 6.2 Hz, 2H, CH_2). ^{13}C NMR (101 MHz, DMSO) δ 171.18, 169.48, 156.49, 143.86, 140.73, 127.65, 127.10, 125.28, 120.12, 65.75, 46.64, 43.30, 40.59. HRMS (ESI): *m/z* calcd for $C_{19}H_{18}N_2O_5+H^+$: 355.1294 [*M*+H]$^+$; found: 355.1294; white lyophilised powder.

H-Ser-OEt·HCl (19)

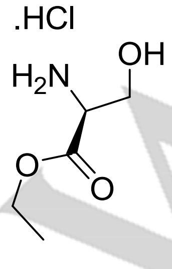

Synthesised via **2.7** from serine in 100% yield. ^{1}H NMR (400 MHz, Deuterium Oxide) δ 4.39 – 4.30 (m, 2H, CH_2), 4.28 (t, *J* = 4.3 Hz, 1H, CH), 4.16 – 4.00 (m, 2H, CH_2), 1.33 (t, *J* = 7.2 Hz, 3H, CH_3). ^{13}C NMR (101 MHz, D_2O) δ 168.42, 63.71, 59.22, 54.72, 13.18. HRMS (ESI): *m/z* calcd for $C_5H_{11}NO_3+H^+$: 134.0817 [*M*+H]$^+$; found: 134.0813; white solid.

Fmoc-Gly-L-Ser-OEt (20)

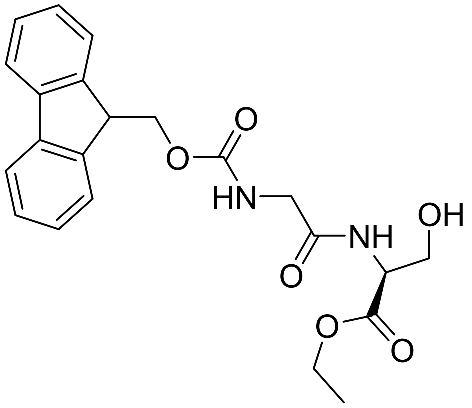

Synthesised via **2.4a** from Fmoc-Gly-OH and **19** in 60% yield. Purified by recrystallization from ethyl acetate : hexane. ^{1}H NMR (400 MHz, Chloroform-*d*) δ 7.74 (d, *J* = 7.6 Hz, 2H, Ar-H), 7.57 (d, *J* = 7.5 Hz, 2H, Ar-H), 7.38 (app. td, *J* = 7.5, 1.1 Hz, 2H, Ar-H), 7.28 (app. td, *J* = 7.5, 1.1 Hz, 2H, Ar-H), 7.20 (d, *J* = 7.6 Hz, 1H, NH), 5.85 (t, *J* = 5.7 Hz, 1H, NH), 4.64 (dt, *J* = 7.3, 3.5 Hz, 1H, CH), 4.38 (d, *J* = 7.1 Hz, 2H, CH_2), 4.23 – 4.15 (m, 3H, CH + CH_2), 4.01 – 3.87 (m, *J* = 5.8 Hz, 4H, 2 x CH_2), 3.38 (br s, 1H, OH), 1.25 (t, *J* = 7.1 Hz, 3H, CH_3). ^{13}C NMR (101 MHz, $CDCl_3$) δ 170.55, 169.70, 157.05, 143.79, 141.37, 127.86, 127.20, 125.17, 120.10, 67.47, 62.87, 62.15, 54.98, 47.11, 44.39, 14.18. HRMS (ESI): *m/z* calcd for $C_{22}H_{24}N_2O_6+H^+$: 413.1713 [*M*+H]$^+$; found: 413.1704; white solid.

Fmoc-Gly-(ψ^{Me,Me}Pro)-L-Ser-OEt (21)

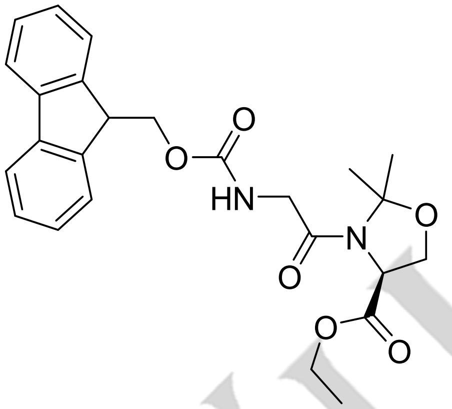

Synthesised via **2.8** from **20** in 40% yield. Purified by flash chromatography, ethyl acetate (30%): hexane over 2 CV, then ethyl acetate (30 – 50%): hexane over 2 CV, ethyl acetate (50%): hexane over 1 CV and then then ethyl acetate (50 – 100%): hexane over 6 CV. ^{1}H NMR (400 MHz, Chloroform-*d*) δ 7.76 (d, *J* = 7.5, 0.9 Hz, 2H, Ar-H), 7.60 (d, *J* = 7.5 Hz, 2H, Ar-H), 7.40 (app. td, *J* = 7.4, 1.2 Hz, 2H, Ar-H), 7.31 (app. td, *J* = 7.4, 1.2 Hz, 2H, Ar-H), 5.71 (t, *J* = 4.6 Hz, 1H, NH), 4.42 – 4.15 (m, 8H, 2 x CH + 3 x CH_2), 3.89 (dd, 2H, CH_2), 1.71 (s, 3H, CH_3), 1.58 (s, 3H, CH_3), 1.31 (t, *J* = 7.2 Hz, 3H, CH_3). ^{13}C NMR (101 MHz, $CDCl_3$) δ 169.76, 165.56, 156.31, 143.99, 141.41, 127.84, 127.20, 125.28, 120.10, 97.25, 67.53, 62.62, 58.62, 47.23, 44.07, 25.16, 23.55, 14.24. HRMS (ESI): *m/z* calcd for $C_{25}H_{28}N_2O_6+H^+$: = 453.2026 [*M*+H]$^+$; found: 453.2021; white brittle powder/sticky oil.

WILEY-VCH

Fmoc-Gly-(ψ[Me,Me]Pro)-L-Ser-OH (22)

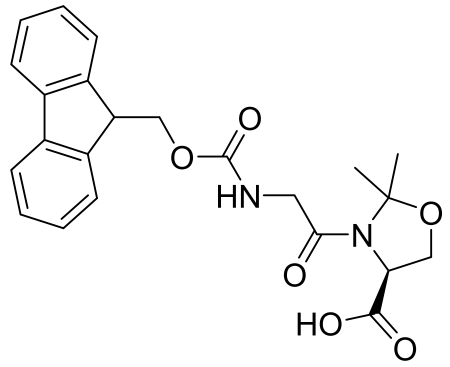

Synthesised via **2.9** from **21** in 70% yield. Purified by flash chromatography, CH_2Cl_2 : methanol (0 – 10%) + acetic acid (0.1 %v/v) over 10 CV. ^{1}H NMR (400 MHz, Chloroform-*d*) δ 8.43 (br s, 1H, CO_2H), 7.75 (d, *J* = 7.7 Hz, 2H, Ar-H), 7.57 (d, *J* = 7.7 Hz, 2H, Ar-H), 7.43 – 7.35 (m, 2H, Ar-H), 7.34 – 7.25 (m, 2H, Ar-H), 6.09 (br m, 1H, NH), 4.55 – 4.14 (m, 6H, 2 x CH, 2 x CH_2), 4.16 – 3.79 (m, 2H, CH_2), 1.72 (s, 3H, CH_3), 1.58 (s, 3H, CH_3). ^{13}C NMR (101 MHz, $CDCl_3$) δ 172.36, 165.29, 157.11, 143.71, 141.41, 127.92, 127.25, 125.23, 120.15, 97.42, 67.95, 58.47, 47.04, 44.22, 25.10, 23.50. HRMS (ESI): *m/z* calcd for $C_{23}H_{24}N_2O_6$+H^+: 425.1713 [*M*+H]$^+$; found: 455.1709; white brittle powder/sticky gum.

Fmoc-Gly-(Dmb)Gly-OH (23)

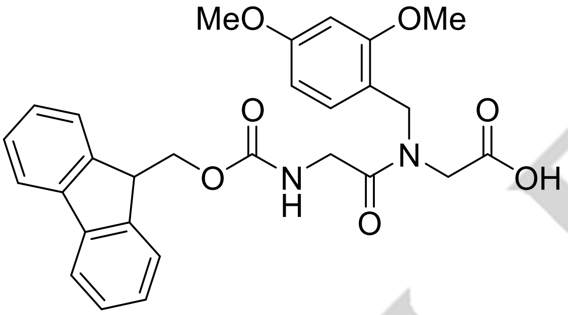

Synthesised via **2.11** from Fmoc-(Dmb)Gly-OH and Fmoc-Gly-OH on 2-chlototrityl chloride resin, in 64% yield. Purified by flash chromatography, CH_2Cl_2 : methanol (0 – 10%) + acetic acid (0.1 %v/v) over 10 CV. ^{1}H NMR (400 MHz, Chloroform-*d*) δ 8.10 (d, *J* = 7.5 Hz, 2H, Ar-H), 7.98 – 7.92 (m, 2H, Ar-H), 7.73 (app. t, *J* = 7.5 Hz, 2H, Ar-H), 7.65 (app. t, *J* = 7.4 Hz, 2H, Ar-H), 7.36 (app. d, *J* = 8.0 Hz, 1H, Ar-H), 6.78 (app d, *J* = 9.8 Hz, 2H, Ar-H), 6.29 (br m, 1H, NH), 4.85 (d, *J* = 62.1 Hz, 2H, CH_2), 4.74 – 4.66 (m, 4H, 2 x CH_2), 4.58 (t, *J* = 7.4 Hz, 1H, CH), 4.40 (d, *J* = 3.2 Hz, 2H, CH_2), 4.15 – 4.09 (m, 6H, 2 x CH_3). ^{13}C NMR (101 MHz, $CDCl_3$) δ 173.15, 169.85, 161.52, 158.95, 156.56, 144.04, 141.41, 130.66, 127.82, 127.21, 125.36, 120.08, 114.94, 104.21, 99.02, 67.35, 55.56, 55.44, 47.25, 47.18, 46.48, 42.79. HRMS (ESI): *m/z* calcd for $C_{28}H_{28}N_2O_7$+H^+: 505.1975 [*M*+H]$^+$; found: 505.1976; white brittle powder/sticky gum.

References

[50] W. C. Powell, K. Johnson, P. Tran, R. Jing, M. A. Walczak, *Synlett* **2024**, *36*, 55.
